# Supplementary material for: Task–technology fit and digital textbook usage outcomes: The mediating role of positive emotion within an S–O–R framework
Source: PLoS One. 2026 Mar 10;21(3):e0344382. doi: 10.1371/journal.pone.0344382 (PMC12974882; doi:10.1371/journal.pone.0344382)
Supplement: S1 File — (DOCX) [file pone.0344382.s001.docx]

# Survey on the Use of Digital English Textbook in Chinese University

Dear Students,

We are conducting a survey on your experience with using digital English textbooks. There are no right or wrong answers, and please respond based on your genuine thoughts and feelings. All responses are anonymous and used solely for academic research purposes. Please feel assured when filling in the questionnaire.

The survey will take approximately 5 minutes. You may exit the questionnaire at any time without giving a reason. If you have any questions or suggestions, please contact us:
Ning Mi, Cunying Fan

Department of College English Teaching, Qufu Normal University

sdmining@qfnu.edu.cn

Thank you for your support and cooperation!

## Part One: Basic Information

1. What is your gender?

- A. Male
- B. Female

2. What is your major?

- A. Biology
- B. Psychology
- C. History
- D. Physics
- E. Statistics
- F. Cyber security

## Part Two: University Students’ Use of Digital English Textbook

Instruction: Please read each statement carefully and indicate how much you agree or disagree with it, based on your true feelings.

### I. Task-Technology Fit

| Statement | Strongly Disagree | Disagree | Neutral | Agree | Strongly Agree |
| --- | --- | --- | --- | --- | --- |
| 1. The English digital textbook is very useful in helping me to complete the unit learning task. | ☐ | ☐ | ☐ | ☐ | ☐ |
| 2. The English digital textbook is very helpful for me to complete the unit learning task. | ☐ | ☐ | ☐ | ☐ | ☐ |
| 3. The English digital textbook makes it very easy to complete the unit learning task. | ☐ | ☐ | ☐ | ☐ | ☐ |
| 4. In general, the digital English textbook meets my English learning needs. | ☐ | ☐ | ☐ | ☐ | ☐ |

### II. Positive Emotion

| Statement | Strongly Disagree | Disagree | Neutral | Agree | Strongly Agree |
| --- | --- | --- | --- | --- | --- |
| 5. I feel active when learning the digital textbook. | ☐ | ☐ | ☐ | ☐ | ☐ |
| 6. I feel happy when learning the digital textbook. | ☐ | ☐ | ☐ | ☐ | ☐ |
| 7. I feel enthusiastic when learning the digital textbook. | ☐ | ☐ | ☐ | ☐ | ☐ |
| 8. I feel excited when learning the digital textbook. | ☐ | ☐ | ☐ | ☐ | ☐ |
| 9. I feel proud when learning the digital textbook. | ☐ | ☐ | ☐ | ☐ | ☐ |
| 10. I feel delighted when learning the digital textbook. | ☐ | ☐ | ☐ | ☐ | ☐ |

### III. Learning Effectiveness

| Statement | Strongly Disagree | Disagree | Neutral | Agree | Strongly Agree |
| --- | --- | --- | --- | --- | --- |
| 11. The English digital textbook has made a positive impact on my view of English learning. | ☐ | ☐ | ☐ | ☐ | ☐ |
| 12. The English digital textbook arouses my interest in English learning. | ☐ | ☐ | ☐ | ☐ | ☐ |
| 13. The English digital textbook encourages me to devote myself to English learning. | ☐ | ☐ | ☐ | ☐ | ☐ |

### IV. Continuance Intention

| Statement | Strongly Disagree | Disagree | Neutral | Agree | Strongly Agree |
| --- | --- | --- | --- | --- | --- |
| 14. I will continuously use the digital English textbook in the future. | ☐ | ☐ | ☐ | ☐ | ☐ |
| 15. I will be more willing to use the digital English textbook in the future. | ☐ | ☐ | ☐ | ☐ | ☐ |
| 16. I will continuously employ the digital English textbook in the future and ramp up its utilization. | ☐ | ☐ | ☐ | ☐ | ☐ |
| 17. I highly recommend others to use the digital English textbook. | ☐ | ☐ | ☐ | ☐ | ☐ |

# **高校数字英语教材使用情况调查问卷**

亲爱的同学：
您好！我们正在进行一项关于数字英语教材使用体验的调查。问卷内容无对错之分，仅用于了解您真实的想法和感受。所有答卷均匿名处理，仅用于学术研究，请您放心填写。

整份问卷大约需要5分钟。您可以在任何时候退出本次问卷，无需说明理由。如果您有任何问题或建议，欢迎联系我们：弭宁,范存英，曲阜师范大学公共外语教学部（sdmining@qfnu.edu.cn）。

感谢您的支持与配合！

**第一部分：基础信息题**

1. 您的性别：
A. 男 B. 女

2. 您的专业：
A. 生物 B. 心理学 C. 历史 D. 物理 E. 统计 F. 网络安全

**第二部分：高校大学生数字英语教材平台使用情况调查**

## 说明：请仔细阅读每个陈述，并指出你在多大程度上同意或不同意它，请根据您的真实感受选择。

## 一、任务与技术匹配度

| 题目 | 非常不同意 | 不同意 | 一般 | 同意 | 非常同意 |
| --- | --- | --- | --- | --- | --- |
| 1.数字英语教材对我完成单元学习任务很有帮助。 | □ | □ | □ | □ | □ |
| 2.数字英语教材对我完成学习任务很有帮助。 | □ | □ | □ | □ | □ |
| 3.数字英语教材让我更容易完成学习任务。 | □ | □ | □ | □ | □ |
| 4.总体来说，数字英语教材能满足我的英语学习需求。 | □ | □ | □ | □ | □ |

## 二、积极情绪

| 题目 | 非常不同意 | 不同意 | 一般 | 同意 | 非常同意 |
| --- | --- | --- | --- | --- | --- |
| 5.学习数字英语教材时，我感到积极主动。 | □ | □ | □ | □ | □ |
| 6.学习数字英语教材时，我感到开心。 | □ | □ | □ | □ | □ |
| 7.学习数字英语教材时，我感到有热情。 | □ | □ | □ | □ | □ |
| 8.学习数字英语教材时，我感到兴奋。 | □ | □ | □ | □ | □ |
| 9.学习数字英语教材时，我感到自豪。 | □ | □ | □ | □ | □ |
| 10.学习数字英语教材时，我感到愉快。 | □ | □ | □ | □ | □ |

## 三、学习成效

| 题目 | 非常不同意 | 不同意 | 一般 | 同意 | 非常同意 |
| --- | --- | --- | --- | --- | --- |
| 11.数字英语教材让我对英语学习有了更积极的看法。 | □ | □ | □ | □ | □ |
| 12.数字英语教材激发了我对英语学习的兴趣。 | □ | □ | □ | □ | □ |
| 13.数字英语教材让我更愿意投入到英语学习中。 | □ | □ | □ | □ | □ |

## 四、持续使用意图

| 题目 | 非常不同意 | 不同意 | 一般 | 同意 | 非常同意 |
| --- | --- | --- | --- | --- | --- |
| 14.我今后会继续使用这个数字英语教材平台。 | □ | □ | □ | □ | □ |
| 15.我会更愿意使用这个数字英语教材平台。 | □ | □ | □ | □ | □ |
| 16.我会继续使用这个数字英语教材平台，并逐步增加使用频率。 | □ | □ | □ | □ | □ |
| 17.我会向他人推荐使用这个数字英语教材平台。 | □ | □ | □ | □ | □ |
