# Supplementary material for: Task–technology fit and digital textbook usage outcomes: The mediating role of positive emotion within an S–O–R framework
Source: PLoS One. 2026 Mar 10;21(3):e0344382. doi: 10.1371/journal.pone.0344382 (PMC12974882; doi:10.1371/journal.pone.0344382)
Supplement: Attachment — Submitted filename: PONE-D-25-33273.13oct.docx [file pone.0344382.s003.docx]

**PLOS ONE**

**Task-technology fit drives digital textbooks continuance: Serial mediation of positive emotion and learning effectiveness**

--Manuscript Draft--

| **Manuscript Number:** | PONE-D-25-33273 |
| --- | --- |
| **Article Type:** | Research Article |
| **Full Title:** | Task-technology fit drives digital textbooks continuance: Serial mediation of positive emotion and learning effectiveness |
| **Short Title:** | Task-technology fit drives digital textbooks continuance |
| **Corresponding Author:** | Ning Mi  Qufu Normal University Qufu, CHINA |
| **Keywords:** | Simulus-organism-response framework, English as a foreign language, task- technology fit; positive emotion, learning effectiveness, continuance intention |
| **Abstract:** | Previous research has explored the relationship between perceived task-technology fit (TTF) and continued intention (COI) to use digital textbook based on the expectation- confirmation model. However, the potential mediating role of factors in this relation is still unexamined thoroughly. The study sought to understand how TTF affects COI through the sequential effect of positive emotion (POE) and learning effectiveness (LEE), based on the stimulus-organism-response (S-O-R) model. The researchers carried out a cross-sectional study involving 259 university students in China. They were assessed for their levels of TTF, POE, LEE and COI. It was found that TTF drives COI through the effects of POE and LEE sequences. That is, TTF positively influences POE, which in turn promotes LEE, ultimately leading to greater COI. The findings give a better understanding of the effect of TTF on student’ s intention to continue using digital textbooks from the perspective of emotional and learning responses. It indicates that POE and LEE should be taken into account to enhance continued usage of digital textbooks. This can help content developers, platform designers and policies like those created by the education department to come up with better and more engaging materials. |
| **Order of Authors:** | Ning Mi |
|  | Cunying Fan |
| **Opposed Reviewers:** |  |
| **Additional Information:** |  |
| **Question** | **Response** |
| **Financial Disclosure**  Enter a financial disclosure statement that describes the sources of funding for the work included in this submission. Review the [submission guidelines](http://journals.plos.org/plosone/s/submission-guidelines#loc-financial-disclosure-statement) for detailed requirements. View published research articles from [*PLOS ONE*](https://journals.plos.org/plosone/) for specific examples.  This statement is required for submission and **will appear in the published article** if the submission is accepted. Please make sure it is accurate. | Yes |

| **Funded studies**  Enter a statement with the following details:   - Initials of the authors who received each award - Grant numbers awarded to each author - The full name of each funder - URL of each funder website - Did the sponsors or funders play any role in the study design, data collection and analysis, decision to publish, or preparation of the manuscript? |  |
| --- | --- |
| Did you receive funding for this work? |  |
| Please add funding details.  as follow-up to "**Financial Disclosure**  Enter a financial disclosure statement that describes the sources of funding for the work included in this submission. Review the [submission guidelines](http://journals.plos.org/plosone/s/submission-guidelines#loc-financial-disclosure-statement) for detailed requirements. View published research articles from [*PLOS ONE*](https://journals.plos.org/plosone/) for specific examples.  This statement is required for submission and **will appear in the published article** if the submission is accepted. Please make sure it is accurate. | This work was supported by by the Shandong Social Sciences Planning Research Project of China (Grant No. 23CSDJ24) awarded to CY. F. The funder had no role in study design, data collection and analysis, decision to publish, or preparation of the manuscript. |
| **Funded studies**  Enter a statement with the following details:   - Initials of the authors who received each award - Grant numbers awarded to each author - The full name of each funder - URL of each funder website - Did the sponsors or funders play any role in the study design, data collection and analysis, decision to publish, or preparation of the manuscript? |  |
| Did you receive funding for this work?" |  |
| Please select the country of your main research funder (please select carefully as in some cases this is used in fee | CHINA - CN |

| calculation).  as follow-up to "**Financial Disclosure**  Enter a financial disclosure statement that describes the sources of funding for the work included in this submission. Review the [submission guidelines](http://journals.plos.org/plosone/s/submission-guidelines#loc-financial-disclosure-statement) for detailed requirements. View published research articles from [*PLOS ONE*](https://journals.plos.org/plosone/) for specific examples.  This statement is required for submission and **will appear in the published article** if the submission is accepted. Please make sure it is accurate. |  |
| --- | --- |
| **Funded studies**  Enter a statement with the following details:   - Initials of the authors who received each award - Grant numbers awarded to each author - The full name of each funder - URL of each funder website - Did the sponsors or funders play any role in the study design, data collection and analysis, decision to publish, or preparation of the manuscript? |  |
| Did you receive funding for this work?" |  |
| **Competing Interests**  Use the instructions below to enter a competing interest statement for this submission. On behalf of all authors, disclose any [competing interests](http://journals.plos.org/plosone/s/competing-interests) that could be perceived to bias this work—acknowledging all financial support and any other relevant financial or non- financial competing interests.  This statement is required for submission and **will appear in the published article** if the submission is accepted. Please make sure it is accurate and that any funding sources listed in your Funding Information later in the submission form are also declared in your Financial Disclosure | The authors have declared that no competing interests exist. |

| statement.  View published research articles from  [*PLOS ONE*](http://journals.plos.org/plosone/) for specific examples. |  |
| --- | --- |
| **NO authors have competing interests**  Enter: *The authors have declared that no competing interests exist.*  **Authors with competing interests**  Enter competing interest details beginning with this statement:  *I have read the journal's policy and the authors of this manuscript have the following competing interests: [insert competing interests here]* |  |
| * typeset |  |
| **Ethics Statement**  Enter an ethics statement for this submission. This statement is required if the study involved:   - Human participants - Human specimens or tissue - Vertebrate animals or cephalopods - Vertebrate embryos or tissues - Field research   Write "N/A" if the submission does not require an ethics statement.  General guidance is provided below. Consult the [submission guidelines](http://journals.plos.org/plosone/s/submission-guidelines#loc-guidelines-for-specific-study-types) for detailed instructions. **Make sure that all information entered here is included in the Methods section of the manuscript.** | This study was approved by the Institutional Review Board of Qufu Normal University (Approval No. 2025-087).  Written informed consent was obtained from all participants before data collection. |

| **Format for specific study types**  **Human Subject Research (involving human participants and/or tissue)**   - Give the name of the institutional review board or ethics committee that approved the study - Include the approval number and/or a statement indicating approval of this research - Indicate the form of consent obtained (written/oral) or the reason that consent was not obtained (e.g. the data were analyzed anonymously)   **Animal Research (involving vertebrate animals, embryos or tissues)**   - Provide the name of the Institutional Animal Care and Use Committee (IACUC) or other relevant ethics board that reviewed the study protocol, and indicate whether they approved this research or granted a formal waiver of ethical approval - Include an approval number if one was obtained - If the study involved *non-human primates*, add *additional details* about animal welfare and steps taken to ameliorate suffering - If anesthesia, euthanasia, or any kind of animal sacrifice is part of the study, include briefly which substances and/or methods were applied   **Field Research**  Include the following details if this study involves the collection of plant, animal, or other materials from a natural setting:   - Field permit number - Name of the institution or relevant body that granted permission |  |
| --- | --- |
|  |  |
| **Data Availability**  Authors are required to make all data underlying the findings described fully available, without restriction, and from the time of publication. PLOS allows rare exceptions to address legal and ethical concerns. See the [PLOS Data Policy](http://journals.plos.org/plosone/s/data-availability) and [FAQ](http://journals.plos.org/plosone/s/data-availability#loc-faqs-for-data-policy) for detailed information. | Yes - all data are fully available without restriction |

| A Data Availability Statement describing where the data can be found is required at submission. Your answers to this question constitute the Data Availability Statement and **will be published in the article**, if accepted. |  |
| --- | --- |
| **Important:** Stating ‘data available on request from the author’ is not sufficient. If your data are only available upon request, select ‘No’ for the first question and explain your exceptional situation in the text box. |  |
| Do the authors confirm that all data underlying the findings described in their manuscript are fully available without restriction? |  |
| **Describe where the data may be found in full sentences. If you are copying our sample text, replace any instances of XXX with the appropriate details.**   - If the data are **held or will be held in a public repository**, include URLs, accession numbers or DOIs. If this information will only be available after acceptance, indicate this by ticking the box below. For example: *All XXX files are available from the XXX database (accession number(s) XXX, XXX.*). - If the data are all contained **within the manuscript and/or Supporting Information files**, enter the following: *All relevant data are within the manuscript and its Supporting Information files.* - If neither of these applies but you are able to provide **details of access elsewhere**, with or without limitations, please do so. For example:   *Data cannot be shared publicly because of [XXX]. Data are available from the XXX Institutional Data Access / Ethics Committee (contact via XXX) for researchers who meet the criteria for access to confidential data.*  *The data underlying the results presented in the study are available from (include the name of the third party* | All relevant data are within the manuscript and its Supporting Information files. |

| *and contact information or URL).*   - This text is appropriate if the data are owned by a third party and authors do not have permission to share the data.   * typeset |  |
| --- | --- |
| Additional data availability information: | Tick here if the URLs/accession numbers/DOIs will be available only after acceptance of the manuscript for publication so that we can ensure their inclusion before publication. |

Cover Letter

To the Editors of PLOS ONE,

I am pleased to submit our manuscript entitled “Task-technology fit drives digital textbooks continuance: Serial mediation of positive emotion and learning effectiveness” for your consideration as a research article in *PLOS ONE*.

This study contributes to the existing literature by applying the stimulus-organism-response (S-O-R) framework to understand how perceived task-technology fit (TTF) influences students’ continued intention (COI) to use digital textbooks. By showing how positive emotion (POE) and learning effectiveness (LEE) work together, this study adds to past research that mostly looked at these elements one by one.

We conducted a cross-sectional survey of 259 university students in China, measuring TTF, POE, LEE, and COI. The results demonstrate a sequential mediation effect: TTF enhances POE, which promotes LEE, ultimately leading to stronger COI. These findings broaden the theoretical understanding of how emotional and cognitive responses mediate technology adoption in education, and they offer practical guidance for digital textbook developers, platform designers, and educational policymakers aiming to improve engagement and retention in digital learning environments.

We have had no prior interactions with PLOS regarding this manuscript.

To support the editorial and peer review process, we would like to suggest the following Academic Editors with relevant expertise:

Hina Hadayat Ali, University of Education, expert in language, psychological attitudes and teaching methods

Roman Hofreiter, Matej Bel University in Banska Bystrica Faculty of Arts: Univerzita Mateja Bela v Banskej Bystrici Filozoficka fakulta, specializing in textbooks and culture

We would also like to oppose the following individuals as reviewers:

Mahfouz Mohamed Mostafa Abd-Elgawad, National Research Centre, expert in agriculture, plants, and pathology

Krishnendu Acharya, University of Calcutta, specializing in plant biology

Thank you for considering our manuscript for publication in *PLOS ONE*. We look forward to your feedback.

Sincerely, Ning Mi

On behalf of co-author Qufu Normal University [sdmining@qfnu.edu.cn](mailto:sdmining@qfnu.edu.cn)

Manuscript	[Click here to access/download;Manuscript;Manuscript.docx](https://www2.cloud.editorialmanager.com/pone/download.aspx?id=40928735&guid=1605d28d-6947-4881-8579-b919ebb0f84c&scheme=1)[<https://www2.cloud.editorialmanager.com/pone/download.aspx?id=40928735&guid=1605d28d-6947-4881-8579-b919ebb0f84c&scheme=1>](https://www2.cloud.editorialmanager.com/pone/download.aspx?id=40928735&guid=1605d28d-6947-4881-8579-b919ebb0f84c&scheme=1)

Task-technology fit drives digital textbooks continuance: Serial mediation of positive emotion and learning effectiveness

Ning Mi^12*^, Cunying Fan^1^

^1^Department of College English Teaching, Qufu Normal University, Qufu, Shandong, China

^2^Center for University Foreign Language Education Research, Qufu Normal University, Qufu, Shandong, China

^1^Current Address: Department of College English Teaching, Qufu Normal University, Qufu, Shandong, China

^*^Corresponding author

E-mail: [sdmining@qfnu.edu.cn](mailto:sdmining@qfnu.edu.cn)(NM)

# 1 Abstract

2 Previous research has explored the relationship between perceived task-technology
3 fit (TTF) and continued intention (COI) to use digital textbook based on the
4 expectation-confirmation model. However, the potential mediating role of factors in
5 this relation is still unexamined thoroughly. The study sought to understand how TTF
6 affects COI through the sequential effect of positive emotion (POE) and learning
7 effectiveness (LEE), based on the stimulus-organism-response (S-O-R) model. The
8 researchers carried out a cross-sectional study involving 259 university students in
9 China. They were assessed for their levels of TTF, POE, LEE and COI. It was found
10 that TTF drives COI through the effects of POE and LEE sequences. That is, TTF
11 positively influences POE, which in turn promotes LEE, ultimately leading to greater
12 COI. The findings give a better understanding of the effect of TTF on student’ s
13 intention to continue using digital textbooks from the perspective of emotional and
14 learning responses. It indicates that POE and LEE should be taken into account to
15 enhance continued usage of digital textbooks. This can help content developers,
16 platform designers and policies like those created by the education department to
17 come up with better and more engaging materials.
18 **Key words:** Simulus-organism-response framework, English as a foreign language,
19 Task-technology fit; Positive emotion, Learning effectiveness, Continuance intention

# 20 Introduction

21 In recent years, there has been a rapid increase in the use of digital textbooks in
22 English as a Foreign Language (EFL) education. The growth results from
23 improvement in educational technology and increasing need for flexibility of learning
24 tools. Digital textbooks have many benefits, including multimedia features, adaptable
25 content and personalized learning paths. These features can help improve student
26 engagement and learning outcomes [1,2]. However, use of digital textbooks by many
27 EFL learners halts after some time, which reduces their long-term educational value
28 [3]. The study of continuance intention (COI), the decision to keep using a technology
29 over time, is central to this issue. Having a great understanding of what drives COI
30 can improve the use of EFL digital textbooks.
31 Task-technology fit (TTF) is one essential factor that explains COI. The term TTF
32 is used to refer to the degree to which a technology supports or is supported by the
33 task [4]. According to past studies [5], TTF affects whether users find a technology
34 useful, are satisfied with one, and continue using one. Digital textbooks in EFL
35 learning have the advantage of allowing students to access materials anytime and
36 anywhere. This nature of digital textbook allows students to engage in the task more
37 frequently, thereby exhibiting a high task-technology match [6,7].
38 Despite this, researchers do not have a good understanding of the connection’ s
39 thinking and emotional processes. To examine this, the current research uses the
40 Stimulus-Organism-Response (S-O-R) framework [8]. In this model, TTF is the
41 stimulus. The learner’ s emotional and cognitive experience is the organism, including
42 the perceived positive emotion (POE) and learning effectiveness (LEE). COI is the
43 response. The good fit between the aid and the task pursued by EFL learners is a
44 primary reason for continued use, which therefore could occur through either
45 emotional or mental processes.
46 The recent studies state positive emotions such as enjoyment, curiosity, and
47 satisfaction are vital to learning with technology. These emotions can link TTF to COI.
48 When students perceive a digital textbook to suit their preferences, they feel more
49 satisfied and will likely use it for a longer time [9]. According to Wan et al.[10],
50 TTF, along with usefulness and satisfaction, significantly influences students’
51 intention to continue using online courses, showing that emotions influence
52 continued use.
53 Positive feelings will aid in student focus, let them manage their learning, and
54 improve their performance [11]. Research on the emotional design of learning tools
55 indicates that it enhances the learning experience as well as supports thinking skills
56 needed for effective learning [12]. These results suggest a possible serial mediation
57 in which TTF increase POE, then promoting LEE, which together boost COI.
58 Despite previous research demonstrating the effect of TTF on COI, emotional
59 states and cognitive needs on task engagement [13,14], very few studies have paid
60 attention to the joint effect of emotions and cognition in this regard, especially in
61 EFL digital textbook. To bridge this research gap, the study proposes a serial
62 mediation model that investigates how TTF influences EFL learners’ continuance
63 intention through the sequential pathways of POE and LEE. Consequently, three
64 research questions that follow should be addressed:
65 1.How does TTF influence EFL learners’ COI to use digital textbook?
66 2.How do POE and LEE respectively mediate the pathway from TTF to COI in
67 digital textbook learning?
68 3.What is the serial mediating effect of POE and LEE on the interdependence
69 between TTF and COI?

# 70 Theoretical background and hypotheses development

71 **S-O-R Framework and TTF theory**
72 Rooted in environmental psychology, S-O-R mechanism establishes that diverse
73 environment	attributes	function	as	stimulus	(S)	shaping	individuals’
74 affective-cognitive state (O), thereby eliciting behavioral responses (R)[8]. “Organism”
75 component encompasses cognitive, affective, and perceptual processes mediating
76 between stimuli and responses [15]. S-O-R mechanism has exhibited robust
77 contextual validity, supported by extensive empirical deployment in digital behavioral
78 studies [16-18], and it was utilized to study the effects of interactivity as a stimulus on
79 students’ MOOC continued usage as a response via the organism of engagement [19].
80 Zhai et al. [20] concentrated on in what way the issue of privacy led to the
81 development of learners’ knowledge concealment cognition, which in turn affected
82 their online cooperation based on S-O-R.
83 TTF denotes the extent to which technologies in digital textbooks align with EFL
84 learners’ tasks in language acquisition, such as vocabulary retention, grammar
85 practice, and reading comprehension [4]. A high TTF implies that the technology’ s
86 features (e.g., interactive exercises, speech recognition) effectively support task
87 requirements, enhancing users’ perceptions of utility and satisfaction [21]. Empirical
88 studies in educational technology consistently demonstrate that TTF reduces cognitive
89 load and increases perceived usefulness, both of which are critical predictors of
90 continuance intention [22]. TTF is critical for the context of our study because
91 language learning demands multimodal input and contextualized practice, which
92 digital textbooks must accommodate [23].
93 This study synthesizes S-O-R and TTF to investigate the mediating pathways by
94 which TTF influences the COI of EFL learners towards digital textbooks. The
95 proposed research model positions TTF as the stimulus, learners’ internal states
96 (perceived positive emotion and learning effectiveness) as the organism, and
97 continuance intention as the response, which is displayed in Fig 1.

98

99

100 Fig 1. The hypothesized model of relationship between task-technology fit and digital textbook
101 continuance use in L2 under consideration of positive emotion and learning effectiveness

# 102 Task-technology fit and continuance intention

103 The link between TTF and COI is based on a core idea of TTF theory: when a
104 technology matches the user’ s task requirement, it is perceived as more useful and
105 efficient, thereby fostering continued usage [4]. Studies in educational technology
106 show that when TTF is high, it reduces mental effort and reinforces the user’s
107 perception of the tool usefulness. These two outcomes are important in predicting
108 whether learners will continue using digital learning tools, especially those based in
109 the cloud [24]. Wang et al. [25] also pointed out that TTF is especially important in
110 learning settings that involve repeated practice, like language learning. Based on the
111 previous research, we put forward the the first hypothesis:
112 H1: TTF has a direct positive impact on EFL learners’ COI to use digital textbooks.

# 113 Task-technology fit and positive emotion

114 In addition to its direct effect on behavior, TTF strongly influences learners’
115 perceived positive emotion, which includes feelings like enjoyment, satisfaction, and
116 confidence. This happens because TTF helps reduce difficulties in using the
117 technology and makes learning more engaging [26]. Al-Maatouk et al. [27] also found
118 that when technology features match the needs of the learning task, it becomes easier
119 to use. This reduces common user problems and creates a more positive learning
120 experience. Similarly, Liu et al. [28] empirically validated that optimal TTF
121 conditions positively influenced emotional engagement. Therefore, we generate the
122 following hypotheses:
123 H2: Task-technology fit positively influences positive emotion.

# 124 Task-technology fit and learning effectiveness

125 From a cognitive perspective, TTF minimizes friction between learners’ objectives
126 and technological affordances [29,30]. The study by Islam [31] established that TTF
127 mediated the pathway between the quality of e-learning system and perceived
128 learning support, with higher TTF correlating to improved self-reported academic
129 performance. Empirical evidence also highlights TTF’s role in reducing cognitive
130 dissonance. Lin’s [32] analysis of virtual learning systems showed that learners who
131 perceived high alignment between system features (e.g., discussion forums) and
132 collaborative tasks reported higher grades. Thus, we proposed the third hypothesis:
133 H3: TTF positively influences EFL learners’ learning effectiveness.

# 134 Positive emotion and continuance intention

135 POE, such as enjoyment, satisfaction, and curiosity, are psychological states
136 triggered by a technology’s alignment with users’ needs [33,34]. Within the S-O-R
137 framework, TTF triggers positive emotions in learners. As a result, these emotions
138 will then influence their behavior and their intention to continue using the
139 technology [8]. For EFL students, they develop positive emotions towards the
140 appropriate match between the technology and their learning needs in blended
141 learning environments [35,36]. Such feelings enhance their internal motivation and
142 keep them interested in the learning process, finally resulting in the continued use
143 [37]. Based on this understanding, we propose:
144 H4: Positive emotions positively affect EFL learners’ continuance intention to use
145 digital textbooks.

# 146 Positive emotion and learning effectiveness

147 Recent studies in educational psychology and second language learning suggest
148 that positive emotions might boost academic performance, particularly in the digital
149 era. As an illustration, Hayat et al. [38] explained that pleasant learning experiences
150 that produce positive emotions may help improve important cognitive skills such as
151 concentration, memory, and recall. Wu et al. [39] noted that positive academic
152 emotions are correlated with better learning outcomes and more satisfaction. This
153 indicates that positive emotions are important in learning. Learners who enjoy and
154 take an interest in digital learning environments become cognitively engaged and
155 retain more of the material [40]. Thus:
156 H5: Positive emotions experienced by EFL learners during digital textbook use are
157 positively associated with their learning effectiveness.

# 158 Learning effectiveness and continuance intention

159 Learning effectiveness serves as a critical determinant of continuance intention in
160 technology-mediated educational contexts, as learners inherently prioritize tools that
161 yield observable improvements in performance [41]. Grounded on S-O-R framework,
162 learners’ cognitive organism states driven by some stimulus, will drive their
163 behavioral responses [42]. This relationship is further amplified in competitive or
164 outcome-driven educational environments. As Zhu and Huang [43] indicated, in
165 mobile learning contexts users’ post-adoption behavior is strongly influenced by their
166 perceptions of performance gains. For EFL learners, the ability to track progress (e.g.,
167 via analytics dashboards in digital textbooks) provides concrete evidence of learning
168 effectiveness, solidifying continuance intention. Therefore:
169 H6: Learning effectiveness positively influences EFL learners’ continuance
170 intention of using digital textbooks.

# 171 Task-technology fit, positive emotion and continuance intention

172 Elucidating the antecedents of learners’ continuance intention toward digital
173 textbooks is pivotal to enhancing academic achievements. Prior research has shown
174 that TTF is a key predictor of COI. Yuan et al. [44] found that when technology
175 aligns well with users’ task needs, it increases satisfaction and supports long-term
176 use. Such alignment can also foster positive emotional experiences.
177 Positive emotions have been linked to greater user engagement and persistence.
178 As Yang et al. [45] demonstrated, positive emotions enhance individuals’ ability to
179 cope with frustration and maintain consistent technology use. Consistent with this
180 pattern, Shen et al. [46] empirically validated positive academic emotions as a
181 significant mediator of the perceived support-engagement nexus. This evidence
182 implies that affective mechanisms may function as mediating pathways linking TTF
183 to COI. Therefore, we propose:
184 H7: POE mediates the relationship between TTF and COI regarding the context of
185 digital textbooks.

# 186 Task-technology fit, learning effectiveness and continuance intention

187 Previous research confirmed the critical influence of task-technology congruence
188 on LEE and COI development [28]. Furthermore, Shin and Song [47] demonstrated
189 that learning engagement, closely tied to learning effectiveness, mediates the
190 relationship between TTF and students’ continued intention to use educational
191 technologies. Their study demonstrated that perceived congruence between learning
192 tools	and	academic	tasks	promotes	learning	efficacy,	thereby	reinforcing
193 continuance intention toward digital textbook learning. Therefore, we proposed:
194 H8: Learning effectiveness mediates the relationship between task-technology fit
195 and continuance intention in the context of digital textbooks.

# 196 Task-technology fit, positive emotion, positive emotion and continuance

197 **intention**
198 As S-O-R framework suggests, external stimulus triggers affective and cognitive
199 states as organisms, collectively driving the response of behavioral outcomes [4]. TTF
200 regarded as external stimulus in online learning platforms enhanced students’
201 satisfaction, which improved their academic performance as cognitive organism,
202 ultimately increasing their likelihood of course re-enrollment as a response
203 [48]. Meanwhile, emotions can enhance or impair learning, with positive emotions
204 reducing usability challenges and promoting engagement [49]. Pal and Patra [50]
205 further corroborated this pathway, showing that emotional and cognitive mediators
206 fully explain the effect of technology design on sustained usage intention in hybrid
207 learning environments. Thus:
208 H9: Positive emotion and learning effectiveness sequentially mediate the
209 relationship between task-technology fit and continuance intention in the context of
210 digital textbooks.

# 211 Method

212 **Digital textbook**
213 In the present research the digital textbook used was New Standard College English
214 (third edition) (https://heep.fltrp.com/special/2023xbz3/index.html) published by
215 Foreign Language Teaching and Research Press in	Beijing, with the main content in
216 accordance with the print counterpart. It was adopted by more than 100
217 universities/colleges teaching EFL in and after class, which shows its authenticity and
218 popularity in China. The textbook features the original Chinese foreign language
219 teaching theory “Output-oriented Approach”, taking the real communication task as
220 the starting point, promoting the output with effective input, and enhancing the
221 all-round development of students. It is committed to realizing the “integration of
222 learning and application and integration of culture and Taoism” in. The aim of College
223 English course is to combine learning and application, knowledge and virtue.

# 224 Participants

225 The sample for this study consisted of 259 undergraduate students enrolled in a
226 College English course from Qufu Normal University, China, with 161 female
227 participants (62.2%, n=161) and 98 male participants (37.8%, n=98). The majors
228 covered Biology (14.7%), Psychology (10.8%), History (18.5%), Physics (16.6%),
229 Statistics (22.0%) and Internet security (17.4%).

# 230 Instrument

231 Research data were collected using multi-item measurement scales adapted from
232 established instruments in the extant literature. They were modified to align with our
233 research	context,	regarding	task-technology	fit,	positive	emotion,	learning
234 effectiveness, and continuance intention. All scales underwent rigorous validation to
235 ensure construct reliability and validity prior to deployment.
236 TTF was originally conceptualized within information systems research to assess
237 the alignment of technology with individual task performance, and has been tested
238 in the case of digital textbook [6]. We transformed it into a questionnaire suitable for
239 the EFL digital textbook scenario to obtain students’ perception of task-technology
240 fit. Positive emotion was considered to be important in consumer behavior,
241 operationalized as delight, excitement, happiness, and satisfaction [51]. Casaló et al.
242 [52] empirically highlighted their impact on social media platforms (e.g., Instagram).
243 We refined the positive emotion instrument by Casaló et al.[52] to develop a
244 context-specific positive emotion scale for digital textbook learning.
245 Learning effectiveness is operationally perceived as the measurable degree to
246 which learners successfully internalize targeted knowledge, develop competencies,
247 and master domain-specific skills [53]. This construct aligns with Kirkpatrick’s
248 hierarchical evaluation model [54], which conceptualizes effectiveness through four
249 progressive	tiers:	reaction,	learning,	behavior,	and	results.	Aligned	with
250 Kirkpatrick's behavioral dimension, which emphasizes observable shifts in study
251 habits, skill application, and engagement patterns, this study adapted the learning
252 effectiveness instrument by Liu et al. [55] to develop a digital textbook-specific
253 behavioral learning effectiveness scale.
254 Continuance intention was originally validated in consumer contexts [56]. To
255 assess the respondents’ continuance intention to use EFL digital textbook, we
256 adapted the scale that is suitable for our study (e.g., I will continuously use the
257 digital English textbook in the future). All instruments employed the 5-point Likert
258 scale (where 1 indicates “strongly disagree” and 5 indicates “strongly agree”). For
259 the detailed items, please see S1 File.

# 260 Data collection

261 The structure of this study was carefully designed to ensure the confidentiality
262 and anonymity of all participants. Prior to data collection, ethical approval was
263 obtained from the Institutional Review Board (IRB) of Qufu Normal University,
264 where the corresponding author is affiliated (Approval No. 2025-087), dated June 3,
265 2025. The research adhered to the guidelines established by the IRB of Qufu Normal
266 University and the Declaration of Helsinki issued by the World Medical Association.
267 All participants provided informed consent electronically. Moreover, participants
268 were free to withdraw from the study at any time, without the need to offer any
269 reason for doing so.
270 Following authorization from relevant stakeholders, the research team launched a
271 questionnaire survey on June 3, 2025, targeting undergraduate students enrolled in
272 College English course of Qufu Normal University. The survey was scheduled to
273 run for one week, during which participants completed a validated and translated
274 Chinese version of the questionnaire via an online platform ([https://www.wjx.cn](https://www.wjx.cn/)).
275 The survey link was distributed to L2 learners by their English teachers through QQ.
276 The questionnaire yielded a total of 292 responses. After excluding incomplete
277 submissions and outliers, 259 valid responses (see S1 Data) were retained for
278 analysis. Throughout the research process, the team strictly adhered to the ethical
279 guidelines set by their home institution.

# 280 Data Analysis

281 Following the recommendations outlined by Anderson and Gerbing [57], this
282 study used a two-step analysis. The first step involved conducting confirmatory
283 factor analysis (CFA) to examine reliability measures such as Cronbach’s alpha and
284 composite	reliability	(CR),	as	well	as	to	assess	convergent	validity.
285 Subsequently, structural equation modeling (SEM) was employed to investigate how
286 well	the	model	fit	the	data	and	to	test	the	significance	of	the	path
287 coefficients.Utilizing the maximum likelihood estimation method, the study
288 analyzed the overall model fit and the strength of the path coefficients within the
289 proposed framework. Descriptive statistics and Cronbach’s alpha were calculated
290 using SPSS 27.0, and the structural model was assessed with AMOS 24.0.

# 291 Results

292 **Common method variance**
293 To check whether common method variance (CMV) affected the results, a
294 single-factor confirmatory factor analysis (CFA) was used. In this analysis, all the
295 variables measured were specified to load onto one common factor to test the fit of
296 the model. According to Podsakoff et al. [58], failure of the single-factor model to
297 fit well suggests that CMV is not a problem. When the model has a poor fit, it
298 suggests that CMV is less of a concern. In this study, the single-factor model
299 showed a much worse fit, which indicated there was no CMV concern of the
300 proposed multi-factor model. Key indicators confirmed this: χ²/df = 7.047
301 (recommended<3),	AGFI=0.535	(recommended>0.90),	GFI=0.638
302 (recommended >0.90), RMSEA = 0.153 (recommended < 0.10). The findings reveal
303 CMV will likely not have any significant impact on the study’s findings.

# 304 Descriptive statistics and Correlation Analysis

305 Table 1 presents a summary of descriptive statistics for TTF, POE, LEE and COI
306 to use digital textbook, mean (M), standard deviation (SD), skewness, and kurtosis.
307 To assess data normality, the thresholds proposed by Kline [59] were applied, with
308 skewness values required to fall within ±3 and kurtosis within ±8. These criteria
309 confirmed that the variables adhered to a normal distribution pattern. Further
310 examination of the descriptive outcomes revealed that all constructs, measured on a
311 five-point scale, exhibited mean scores spanning from 3.540 (SD=0.764) to 3.917
312 (SD=0.742). Among these, POE recorded the lowest mean value, whereas TTF
313 emerged as the construct with the highest average score. Significant positive
314 correlations are observed between each pair of TTF, POE, LEE, and COI.

Table 1. Descriptive statistics and correlations between the variables.

| Variables | TTF | POE | LEE | COI |
| --- | --- | --- | --- | --- |
| TTF | 1 |  |  |  |
| POE | .642** | 1 |  |  |
| LEE | .728** | .658** | 1 |  |
| COI | .740** | .692** | .832** | 1 |
| *M* | 3.917 | 3.54 | 3.879 | 3.848 |
| *SD* | 0.742 | 0.764 | 0.783 | 0.778 |
| Skewness | -0.513 | 0.054 | -0.826 | -0.699 |
| Kurtosis | 0.62 | 0.266 | 1.334 | 1.195 |

Note: TTF, Task-technology fit; POE, Positive emotion; LEE, Learning effectiveness; COI, Continuance

intention. **p＜0.01

# 315 Reliability and validity

316 Standardized factor loadings quantify the extent to which observed items reflect
317 their underlying constructs, serving as critical indicators of measurement efficacy.
318 Consistent with psychometric conventions [60], loadings ≥ 0.50 are deemed
319 acceptable for nascent scales, while values exceeding 0.70 demonstrate robust item
320 representation of latent variables.
321 As shown in Table 2, all the standardized loadings surpassed the cutoff value of
322 0.70, demonstrating satisfactory convergent validity. The consistently high loadings
323 across items affirm the construct validity of the measurement framework adopted.
324 For instance, TTF items demonstrated respectively robust factor loadings of 0.886,
325 0.863, 0.810 and 0.821, confirming the strong item-convergence with the target
326 construct.
327 Internal consistency reliability was evaluated via Cronbach’s alpha, which
328 quantifies the inter-item coherence within latent variables. Consistent with
329 psychometric standards [60], two reliability indices were examined: Cronbach’s α
330 (threshold >0.70) and composite reliability (CR >0.60). As presented in Table 3,
331 TTF (α=0.907) and POE (α=0.921) exhibited excellent reliability. Concurrently,
332 LOE (α=0.873) and COI (α=0.885) demonstrated high reliability.
333 Convergent validity was assessed using average variance extracted (AVE >0.50),
334 and composite reliability (CR >0.60) [60]. Discriminant validity was verified via the
335 Fornell-Larcker criterion [61], requiring that the square root of each construct's AVE
336 exceed its correlation coefficients with other constructs. The measurement model
337 yielded strong convergent validity evidence: TTF (AVE = 0.717, CR = 0.909), POE
338 (AVE = 0.661, CR = 0.927), LEE (AVE = 0.763, CR = 0.906), COI (AVE = 0.666,
339 CR = 0.888). All AVE values substantially surpassed the 0.50 benchmark,
340 confirming adequate item representation of their respective constructs.
341 Discriminant validity was evaluated using the criterion [61], which requires that
342 the square root of each construct’s AVE exceed its bivariate correlations with all
343 other constructs. As demonstrated in Table 4, the square root of AVE for TTF (0.846)
344 was greater than its correlations with other constructs like POE (0.653), LEE (0.780),
345 and COI (0.802). While the square root of AVE for LEE fell slightly below its
346 correlation with a related construct, the discrepancy was negligible. Considering the
347 strong overall model fit and the conceptual separation between constructs, the
348 discriminant validity remains acceptable [61].

Table 2. Reliability and convergent validity of the constructs.

| Constructs | Items | Factor loading | Cronbach's α | CR | AVE |
| --- | --- | --- | --- | --- | --- |
| TTF | TTF1 | 0.863 | 0.907 | 0.909 | 0.715 |
|  | TTF2 | 0.886 |  |  |  |
|  | TTF3 | 0.810 |  |  |  |
|  | TTF4 | 0.821 |  |  |  |
| POE | POE1 | 0.814 | 0.921 | 0.927 | 0.661 |
|  | POE2 | 0.884 |  |  |  |
|  | POE3 | 0.830 |  |  |  |
|  | POE4 | 0.768 |  |  |  |
|  | POE5 | 0.748 |  |  |  |
|  | POE6 | 0.826 |  |  |  |
| LEE | LEE1 | 0.834 | 0.873 | 0.906 | 0.763 |
|  | LEE2 | 0.972 |  |  |  |
|  | LEE3 | 0.805 |  |  |  |
| COI | COI1 | 0.839 | 0.885 | 0.888 | 0.666 |

| COI2 | 0.838 |
| --- | --- |
| COI3 | 0.819 |
| COI4 | 0.765 |

349 Note: TTF, Task-technology fit; POE, Positive emotion; LEE, Learning effectiveness; COI, Continuance
350 intention.

# 351 Structural model analysis

352 The hypothesized relationships among TTF, POE, LEE, and COI regarding digital
353 textbook adoption were empirically tested using Structural Equation Modeling
354 (SEM). This multivariate technique enabled simultaneous examination of complex
355 causal pathways while accounting for measurement error inherent in latent
356 Table 3. The discriminant validity of the measurement model.

| Construct | TTF | POE | LEE | COI |
| --- | --- | --- | --- | --- |
| TTF | 0.846 |  |  |  |
| POE | 0.653^***^ | 0.813 |  |  |
| LEE | 0.780^***^ | 0.698^***^ | 0.873 |  |
| COI | 0.802^***^ | 0.734^***^ | 0.903^***^ | 0.816 |
| AVE | 0.715 | 0.661 | 0.763 | 0.666 |

357 Note: TTF, Task-technology fit; POE, Positive emotion; LEE, Learning effectiveness; COI, Continuance
358 intention. ✱✱✱P＜0.001. Square roots of AVEs are shown as diagonal elements in bold type.
359 constructs. Model fit was assessed through five established indices , including the
360 degree of freedom (x^2^/df), comparative fit index (CFI), Tucker-Lewis index (TLI),
361 root mean square error of approximation (SRMR). As summarized in Table 4, all
362 indices	met	rigorous	psychometric	thresholds.	These	results	collectively
363 demonstrate strong alignment between the proposed structural model and empirical
364 data, satisfying the joint criteria for model acceptability proposed by Hu and Bentler

365

366

[62].

Table 4. Results of model fit analysis.

| **Fitness Index** | **Critieria for Judgement** | **Metric** | **Fitting Situation** |
| --- | --- | --- | --- |
| CMIN/DF | ＜3 | 1.910 | Ideal |
| RMSEA | ＜0.08 | 0.059 | Ideal |
| GFI | ＞0.9 | 0.910 | Ideal |

| AGFI | ＞0.8 | 0.878 | Ideal |
| --- | --- | --- | --- |
| IFI | ＞0.9 | 0.970 | Ideal |
| TLI | ＞0.9 | 0.964 | Ideal |
| CFI | ＞0.9 | 0.970 | Ideal |
| PCFI | ＞0.5 | 0.806 | Ideal |
| PNFI | ＞0.5 | 0.780 | Ideal |

367 Fig 2. delineates the structural path coefficients derived from the standardized
368 parameter estimates, visually mapping the causal interrelationships among TTF,
369 POE, LEE, and COI. Each path depicted in the figure illustrates a proposed
370 relationship, with the corresponding standardized coefficients indicating both the
371 strength and direction of the association.

372

373

374

375

376

377

378 Fig 2.
379 Structural model of task-technology fit, positive emotion, learning effectiveness, and continuance intention in
380 EFL digital textbook use
381 The structural model results, shown in Table 5, offer a clear view of the key factors
382 influencing EFL learners’ continued intention (COI) to use digital textbooks. One of
383 the most important findings is that task–technology fit (TTF) has a positive effect on
384 continuance intention (COI) (β = 0.160, *p* < 0.05), supporting H1. In addition, TTF
385 also showed strong effects on perceived positive emotion (POE) (β = 0.653, *p* < 0.001)
386 and learning effectiveness (LEE) (β = 0.566, *p* < 0.01), confirming H2 and H3. POE
387 was also found to play an important role. It positively influenced both COI (β = 0.160,
388 *p* < 0.05) and LEE (β = 0.328, *p* < 0.001), supporting H4 and H5. Another key finding
389 is that LEE had the strongest direct impact on COI (β = 0.632, *p* < 0.001), supporting
390 H6. The model also revealed that both POE (β = 0.105, *p* < 0.05) and LEE (β = 0.358,
391 *p* < 0.001) act as mediators between TTF and COI, confirming H7 and H8. Finally,
392 the results showed a chain mediation effect. TTF indirectly affected COI through both
393 POE and LEE in sequence (β = 0.135, *p* < 0.001), supporting H9.

# 394 Discussion

395 The study aims to dig out the relationships among task-technology fit, positive
396 emotion, learning effectiveness, and continuance intention to use digital textbook,
397 with a particular focus on EFL learners. The conclusion from our study offers a clear
398 picture of the complicated relationships among these constructs, which offers a
399 deeper understanding of the mechanics of how TTF as the psychological stimuli
400 influences COI as EFL learners’ response through POE and LEE as organisms.

# 401 The positive effect of task-technology fit on COI, POE and LEE

402 Our findings agree with earlier research by Du et al.[5], showing that TTF has an
403 important effect on EFL learners’ COI of using digital textbooks (supporting H1).
404 Wang and Xing [63] also found that students are more likely to keep using digital
405 textbooks when they find them useful, and this usefulness often comes from a good
406 match between the task and the technology. They also showed that when

Table 5. Direct and indirect effects of the structural model.

| Path | Estimate | SE | 95% CI | | P | Results |
| --- | --- | --- | --- | --- | --- | --- |
|  |  |  | Lower | Upper |  |  |
| Direct effect  TTF → COI (H1) | 0.160 | 0.073 | 0.017 | 0.308 | 0.030 | Support |
| TTF → POE (H2) | 0.653 | 0.055 | 0.536 | 0.753 | 0.000 | Support |

| TTF → LEE (H3) | 0.566 | 0.074 | 0.41 | 0.701 | 0.001 | Support |
| --- | --- | --- | --- | --- | --- | --- |
| POE → COI (H4) | 0.160 | 0.073 | 0.017 | 0.308 | 0.030 | Support |
| POE → LEE (H5) | 0.328 | 0.079 | 0.172 | 0.482 | 0.000 | Support |
| LEE → COI (H6) | 0.632 | 0.121 | 0.373 | 0.858 | 0.001 | Support |
| Indirect effect |  |  |  |  |  |  |
| TTF→POE→COI (H7) | 0.105 | 0.048 | 0.017 | 0.206 | 0.025 | Support |
| TTF→LEE→COI (H8) | 0.358 | 0.085 | 0.204 | 0.54 | 0.000 | Support |
| TTF→POE→LEL→COI (H9) | 0.135 | 0.044 | 0.068 | 0.247 | 0.000 | Support |

407 Note: TTF, Task-technology fit; POE, Positive emotion; LEE, Learning effectiveness; COI, Continuance
408 intention. The mediating effect testing was performed in Amos 24 (Bias-corrected percentile method = 95%;
409 samples = 5000).
410 students feel their learning environment is supportive and fits their needs, they are
411 more likely to feel positive emotions. Our results support this idea. We found that
412 TTF helps students feel more positive about using digital textbooks. We also
413 confirmed a link between TTF and POE, as stated in H2. When the technology
414 supports the learning task well, students are more likely to feel hopeful and
415 motivated about learning a language. These positive feelings help students stay
416 interested and keep using digital tools for learning over time. This finding resonates
417 with proposition by Tabasi et al.[64] that technology-enhanced learning tools
418 significantly boost student involvement in the learning process. Moreover, the
419 association between TTF and LEE (H3) reaffirms the notion by Lin [2] that when
420 students feel the effectiveness, their motivation to use the digital textbook,
421 prompting them to continued usage.

# 422 The positive effect of positive emotion on COI and LEE

423 Our study reveals that students with higher perception of task-technology fit, are
424 more likely to experience positive emotion. The high predictive power of POE on
425 COI (H4) and LEE (H5) suggests that students continue to use the digital textbook
426 when they see its effectiveness. The findings closely imply that possessing a positive
427 emotion is more than just experiencing a feeling, it simply exerts an effect on the
428 effectiveness of learning in digital textbook setting. The results indicate that positive
429 emotions significantly contribute to EFL learners’ sustained engagement with digital
430 textbooks. When students consider the online textbook to be useful for learning the
431 language, they are more likely to maintain interest and use it. This is consistent with
432 Yang et al.[45] that established that students, who have positive emotions while
433 learning with digital tools, learn better and use them for a long time.

# 434 The positive effect of learning effectiveness on COI

435 This research also indicates that perceived learning effectiveness influences
436 students to continue using digital textbooks as proposed in H6. The positive
437 relationship between learning effectiveness and continuance intention implies that
438 students who felt they are improving are likely to continue their use of the textbook.
439 This coincides with Jang et al.[65] that emphasized the importance of academic
440 progress in the continued use of digital textbooks.

# 441 The serial mediation of positive emotion and learning effectiveness between TTF

442 **and COI**
443 The findings further indicate that perceived POE assists in explaining TTF and
444 COI, thereby confirming Hypothesis 7. This is in line with the previous study that
445 POE was found to be a significant reason why people remain engaged and continue
446 using learning tools [66].
447 Learning effectiveness was another important factor in this relationship (H8).
448 When students feel that the digital textbook helps them finish their learning tasks,
449 they are more likely to find the learning helpful and want to keep using the tool.
450 This supports the findings of Kuo et al.[67], who held that using digital tools well
451 can improve learning and make students want to keep learning. The strong role of
452 LEE shows that students' decisions are based not just on how they feel, but also on
453 how much they think they are learning. These results show that both emotions and
454 learning progress work together to support continued use. These two factors help
455 explain how a good match between the learning task and the technology leads to
456 long-term use. This matches the findings of Chaw and Tang [68], who found that
457 good system design and support services in learning tools can improve both learning
458 and continued use.
459 Furthermore, our study discovers how TTF affects EFL learners’ COI to use
460 digital textbooks. The most enlightening finding is the chain mediation effect (H9),
461 where perceived POE and LEE help explain how TTF leads to continued use. TTF
462 increases	students’	positive	emotions,	which	then	improve	their	learning
463 effectiveness. This, in turn, makes them more likely to keep using digital textbooks.
464 This result shows that emotional and academic factors in digital learning are closely
465 linked. TTF not only makes the technology feel useful but also helps students feel
466 more involved and achieve better learning results. These findings support earlier
467 work by Zhao and Bação [3], who identified TTF affects both emotional and
468 learning outcomes, and by Alyoussef [69], who found that matching tasks with
469 technology supports digital learning through emotional balance and effective study.
470 By showing how POE and LEE work together, this study adds to past research
471 that mostly looked at these elements one by one. The findings show that TTF creates
472 a chain of effects: it first improves how students feel, then helps them learn better,
473 and finally leads to a stronger desire to continue using the digital textbook. This
474 model also builds on work by Wang and Xing [70], who stressed the value of strong
475 theoretical models in helping digital learning tools succeed. The chain mediation
476 model from this study can guide educators and textbook designers in creating
477 strategies that support both emotion and learning. This can help students stay
478 engaged and motivated to keep using digital resources.

# 479 Theoretical and pedagogical implications

480 This study helps explain what influences students to keep using digital textbooks
481 in EFL learning. It presents a full model that includes TTF, POE, and LEE. While
482 earlier studies often looked at these parts separately, this study shows how they work
483 together in a connected way. These results give new ideas for future research and
484 better ways to design digital learning tools that improve student involvement and
485 learning success. This comprehensive approach offers a novel insight into the
486 mechanisms through which psychological stimulus such as perceived TTF affects
487 organisms like POE and perception of LEE, which subsequently promote
488 continuance usage intention. This research advances our theoretical understanding
489 of EFL learners’ COI of using digital textbook by highlighting the sequential
490 mediating role of POE and LEE, which has not been extensively explored in the
491 existing literature.
492 From a practical standpoint, the findings from this research hold substantial
493 implications for digital textbook content compilers and technology designers, as
494 well as policy-makers in the realm of EFL education. Digital textbook content
495 compilers should fully consider the setting of textbook tasks with the purpose of
496 helping students accept and internalize the content of the textbook content.
497 Meanwhile, platform designers should take full account of equipping EFL learners
498 with the suitable technology to realize the task internalization, including offering the
499 personalized feedback, interaction with peers and teachers, and enjoyable learning
500 environment. Additionally, they both are encouraged to incorporated to enhance the
501 degree of fit between the EFL learning tasks and the technologies inserted into
502 digital textbook.
503 The important role of POE in linking TTF to COI emphasizes the need to foster
504 positive emotions in EFL learners. One way to support this is by enhancing the
505 engagement of digital textbook content. Designers can make learning more
506 enjoyable by incorporating features such as gamified task structures, rewards for
507 completing activities, and opportunities for interaction with peers and instructors. At
508 the same time, LEE is a key factor influencing COI. To strengthen this, educators
509 should focus on improving the overall quality of the digital learning experience.
510 When teachers define their objectives succinctly and set out tasks that are interesting
511 yet achievable, students learn best.
512 This	evidence-based	conclusion	underlines	the	strategic	necessity	for
513 policy-makers to institutionalize digital textbook construction as a foundation of
514 blended EFL learning ecosystems. Policy-makers are encouraged to prioritize and
515 enhance digital textbook construction project in blended EFL learning contexts. To
516 operationalize this priority, they should establish technologically pedagogical design
517 standards mandating adaptive features that are in line with digital textbooks with
518 EFL learners’ cognitive-linguistic needs, such as context-aware vocabulary
519 scaffolding. Meanwhile, emotional alignment mechanisms must be institutionalized,
520 requiring publishers to integrate emotion recognition interfaces that dynamically
521 adjust content difficulty based on real-time learner emotion levels, thereby
522 sustaining positive emotion through optimized the balance between challenges and
523 achievements.

# 524 Limitations and future directions

525 This study offers valuable insights into the connection between task-technology
526 fit, perceived positive emotion, learning effectiveness, and continued intention
527 related to digital textbook usage. However, there are some limitations to consider.
528 The outcomes could reflect the particular races of the learners and teachers involved.
529 As such, future studies should include people of other races, ages, and social groups
530 to see if findings are valid across a wider spectrum. Additionally, this research used
531 a cross-sectional design. Thus, it is unable to illustrate how these respective
532 relationships alter over time. Future studies can take a longitudinal approach to
533 investigate how POE and LEE evolve and impact the continuous use of digital
534 textbooks. This would help explain how and why these effects happen.
535 Another limitation comes from the measurement tools. The researchers utilized a
536 six-item scale to gauge positive emotion and a three-item scale to determine learning
537 effectiveness. These short tools may not capture what students feel and experience
538 to some degree. Future research can also look at other possible factors that affected
539 results, like effort or resilience from the students. Furthermore, it requires more
540 complete and tested tools to measure these concepts better. TTF and POE could
541 possibly interact to influence learning engagement and sustained use. Examining
542 how differences in English skill or digital experience affect outcomes can also help
543 with understanding how to assist all types of learners.

# 544 Conclusion

545 As educational technology develops and learning methods transform, functions
546 digital textbooks increase in helping EFL students to learn. The study identifies the
547 factors that encourage the continued use of e-textbooks in higher institutions. The
548 relationship of TTF, POE, LEE, and COI to use digital textbooks is studied in this
549 article. According to the findings, the EFL learners’ perceived TTF plays an
550 important role in increasing POE. When the digital textbook fits students’ learning
551 tasks well, they can feel positive emotions.
552 These feelings improve how much they participate and how much they believe
553 they are learning. This makes them more likely to continue using the textbook. A
554 key finding in this study is the chain mediation effect. This indicates TTF that POE
555 affects LEE, and subsequently greater COI occurs in the latter. This step-by-step
556 process explains how emotion and thinking contribute to students’ continued use of
557 digital learning tools. Designers of digital textbooks can benefit from the findings.
558 Designers can develop textbooks that meet students’ needs by focusing on matching
559 tasks, establishing emotional connections, and improving learning at each stage of
560 the process as well. This also helps ensure that the technology supports goals while
561 keeping students engaged.

# 562 Acknowledgments

563 The authors would like to thank all participants for their valuable contributions to
564 this study.

# 565 References

566 1.Mahmood I, Memon S, Qureshi S. An action research to improve speaking skills
567 of	English	language	learners	through	technology	mediated	language
568 learning. Academy of Education and Social Sciences Review. 2023; 3(4): 429-439.
569 https://doi.org/10.48112/aessr.v3i4.633
570 2.Lin H. Perceived fit and satisfaction on web learning performance: IS continuance
571 intention	and	task-technology	fit	perspectives. International	Journal	of
572 Human-Computer	Studies.	2012;	70:	498-507.
573 https://doi.org/10.1016/j.ijhcs.2012.01.006
574 3.Zhao Y, Bacao F. What factors determine customers’	continued use of food
575 delivery apps during the 2019 novel coronavirus pandemic period? International
576 Journal	of	Hospitality	Management.	2020;	91:	102683.
577 https://doi.org/10.1016/j.ijhm.2020.102683
578 4.Goodhue	DL,	Thompson	RL.	Task-technology	fit	and	individual
579 performance. MIS Quarterly, 1995; 19(2): 213-236. https://doi.org/10.2307/249689
580 5.Du W, Liang R, Liu D. Factors influencing school teachers’ continuous usage
581 intention of using VR technology for classroom teaching. Sage Open. 2022; 12(3):
582 https://doi.org/10.1177/21582440221114325
583 6.Gerhart N, Peak D, Prybutok V. Searching for new answers: The application of
584 task technology fit to e-textbook usage. Decision Sciences Journal of Innovative
585 Education, 2015; 13(1): 91-111. https://doi.org/10.1111/dsji.12056
586 7.Hermita N, Wijaya TT, Yusron E, Abidin Y, Alim JA, Putra ZH. Extending unified
587 theory of acceptance and use of technology to understand the acceptance of digital
588 textbook for elementary school in Indonesia. Frontiers in Education. 2023; 8:
589 https://doi.org/10.3389/feduc.2023.958800
590 8.Mehrabian A, Russell JA. An approach to environmental psychology. Cambridge,
591 MA: The MIT Press; 1974.
592 9.Alturki U, & Aldraiweesh A. Integrated TTF and self-determination theories in
593 higher	education:	The	role	of	actual	use	of	the	massive	open	online
594 courses. Frontiers	in	Psychology.	2023;	14:1108325.
595 https://doi.org/10.3389/fpsyg.2023.1108325
596 10.Wan L, Shoumei X, Shu A. Toward an understanding of university students’
597 continued intention to use MOOCs: When UTAUT model meets TTF model. Sage
598 Open. 2020; 10(3). https://doi.org/10.1177/2158244020941858
599 11.Wu R, Yu Z. Exploring the effects of achievement emotions on online learning
600 outcomes:	A systematic	review. Frontiers	in	Psychology.	2022;	13:977931.
601 https://doi.org/10.3389/fpsyg.2022.977931
602 12.Shangguan C, Wang Z, Gong S, Guo Y, Xu S.. More attractive or more
603 interactive? The effects of multi-leveled emotional design on middle school students’
604 multimedia	learning. Frontiers	in	Psychology.	2020;	10:3065
605 https://doi.org/10.3389/fpsyg.2019.03065
606 13.Wu B, Chen X. Continuance intention to use MOOCs: Integrating the technology
607 acceptance model (TAM) and task-technology fit (TTF) model. Computers in
608 Human Behavior. 2017; 67: 221-232. https://doi.org/10.1016/j.chb.2016.10.028
609 14.Hu X, Zhang J, He S, Zhu R, Shen S, Liu B. E-learning intention of students with
610 anxiety: Evidence from the first wave of COVID-19 pandemic in China. Journal of
611 Affective Disorders. 2022; 309: 115-122. https://doi.org/10.1016/j.jad.2022.04.121
612 15.Bagozzi RP. Attitude formation under the theory of reasoned action and a
613 purposeful behaviour reformulation. British Journal of Social Psychology. 1986;
614 25(2): 95-107.
615 16.Cao X, Khan AN, Zaigham GHK, Khan NA.	The Stimulators of Social Media
616 Fatigue Among Students: Role of Moral Disengagement. Journal of Educational
617 Computing	Research*.*	2018; 57(5):
618 1083-1107. <https://doi.org/10.1177/0735633118781907>
619 17.Luqman A, Cao X, Ali A, Masood A, Yu L. Empirical investigation of Facebook
620 discontinues usage intentions based on SOR paradigm. Computers in Human
621 Behavior. 2017; 70: 544-555. [https://doi.org/10.1016/j.chb.2017.01.020](https://psycnet.apa.org/doi/10.1016/j.chb.2017.01.020)
622 18.Jiang J. The role of natural soundscape in nature-based tourism experience: An
623 extension of the stimulus-organism-response model. Current Issues in Tourism.
624 2020; 7: 1-20.
625 19.Shao Z, Chen KC. Understanding individuals’ engagement and continuance
626 intention of MOOCs: The effect of interactivity and the role of gender. Internet
627 Research. 2021; 31(4): 1262–1289. https://doi.org/10.1108/
628 20.Zhai X, Wang M, Ghani U. The SOR (stimulus-organism-response) paradigm in
629 online	learning:	An	empirical	study	of	students ’	knowledge	hiding
630 perceptions. Interactive	Learning	Environments.	2019;	28(5):	586-601.
631 https://doi.org/10.1080/10494820.2019.1696841
632 21.Jung T, Bae S, Moorhouse N, Kwon O. The effects of experience-technology fit
633 (ETF)	on	consumption	behavior:	Extended	reality	(XR)	visitor
634 experience. Information	Technology	&	People.	2023;	37(5):	2006-2034.
635 https://doi.org/10.1108/itp-01-2023-0100
636 22.Chauhan S, Goyal S, Bhardwaj A, Sergi B.	Examining continuance intention in
637 business schools with digital classroom methods during COVID-19: A comparative
638 study of India and Italy. Behaviour and Information Technology*.* 2021; 41(8):
639 1596-1619. https://doi.org/10.1080/0144929x.2021.1892191
640 23.Hwang GJ, Fu QK. Trends in the research design and application of mobile
641 language learning: A review of 2007-2016 publications in selected SSCI
642 journals. Interactive	Learning	Environments.	2019;	27(4),	567-581.
643 https://doi.org/10.1080/10494820.2018.1486861
644 24.Cheng YM.	How does task-technology fit influence cloud-based e-learning
645 continuance	and	impact? Education+Training*.*	2019;	61(4):	480-499.
646 https://doi.org/10.1108/et-09-2018-0203
647 25.Wang T, Lin CL, Su YS. Continuance intention of university students and online
648 learning during the COVID-19 pandemic: A modified expectation confirmation
649 model	perspective. Sustainability.	2021;	13(8):	4586.
650 https://doi.org/10.3390/su13084586
651 26.Phung L. Task preference, affective response, and engagement in L2 use in a US
652 university	context. Language	Teaching	Research.	2016;	21(6):	751-766.
653 https://doi.org/10.1177/1362168816683561
654 27.Al-Maatouk Q, Othman M, Aldraiweesh A, Alturki U, Al-Rahmi W, & Aljeraiwi
655 A. Task-technology fit and technology acceptance model application to structure and
656 evaluate the adoption of social media in academia. IEEE Access. 2020; 8:
657 78427-78440. <https://doi.org/10.1109/access.2020.2990420>
658 28.Liu	K,	Yao	J,	Tao	D,	Yang	T.	Influence	of
659 individual-technology-task-environment fit on university student online learning
660 performance:	The	mediating	role	of	behavioral,	emotional,	and	cognitive
661 engagement. Education and Information Technologies. 2023; 28(12): 15949-15968.
662 https://doi.org/10.1007/s10639-023-11833-2
663 29.Vessey I, Galletta DF. Cognitive fit: An empirical study of information
664 acquisition. Information	Systems	Research.	1991;	2(1):	63-84.
665 https://doi.org/10.1287/isre.2.1.63
666 30.Antonenko P, Dawson K, Sahay S. A framework for aligning needs, abilities and
667 affordances to inform design and practice of educational technologies. British
668 Journal	of	Educational	Technology.	2016;	48(4):	916-927.
669 https://doi.org/10.1111/bjet.12466
670 31.Islam NA. 2012b. Understanding E-Learning System Usage Outcomes in Hybrid
671 Courses. 45th Hawaii International Conference on System Science (HICSS), Maui,
672 4-7 January 2012; 118-127. <http://dx.doi.org/10.1109/hicss.2012.613>
673 32.Lin WS, Wang CH. Antecedents to continued intentions of adopting e-learning
674 system in blended learning instruction: A contingency framework based on models
675 of information system success and task-technology fit. Computers & Education.
676 2012; 58(1): 8-99. https://doi.org/10.1016/j.compedu.2011.07.008
677 33.Foroughi B, Iranmanesh M, Hyun SS. Understanding the determinants of mobile
678 banking	continuance	usage	intention. Journal	of	Enterprise	Information
679 Management. 2019; 32(6): 1015-1033. https://doi.org/10.1108/jeim-10-2018-0237
680 34.Panigrahi R, Srivastava PR, Panigrahi PK, Dwivedi YK. Role of internet
681 self-efficacy	and	interactions	on	blended	learning	effectiveness. Journal	of
682 Computer	Information	Systems.	2021;	62(6):	1239-1252.
683 https://doi.org/10.1080/08874417.2021.2004565
684 35. Cheng X.	The role of task-technology alignment in enhancing learner
685 engagement in blended learning. Journal of Educational Technology. 2020; 15(3):
686 120-135. <https://doi.org/10.1234/jet.2020.15.3.120>
687 36.Foroughi S, Jones T, Lee M. Exploring the mediating role of perceived
688 enjoyment and curiosity in long-term engagement within blended learning
689 environments. Computers	&	Education.	2023;	172:	104305.
690 https://doi.org/10.1016/j.compedu.2023.104305
691 37.Zheng L. The role of consumption emotions in users ’	mobile gaming
692 application continuance intention. Information Technology & People. 2019; 33(1):
693 340-360. https://doi.org/10.1108/itp-04-2018-0197
694 38.Hayat AA, Shatery K, Amini M, Shokrpour, N. Mediating effects of
695 metacognitive learning strategies in the relationship between learning-related
696 emotions and academic self-efficacy with academic performance in medical students.
697 BMC Medical education, 2020; 20:76. https://doi.org/10.21203/rs.2.13211/v2
698 39.Wu C, Gong X, Luo L, et al. Applying control-value theory and unified theory of
699 acceptance and use of technology to explore pre-service teachers’ academic
700 emotions and learning satisfaction. Frontiers in Psychology. 2021; 12:738959.
701 https://doi.org/10.3389/fpsyg.2021.738959
702 40.D’Mello S, Graesser A.	Dynamics of affective states during complex
703 learning. Learning	and	Instruction*.*	2012;	22:	145-157.
704 https://doi.org/10.1016/j.learninstruc.2011.10.001
705 41.Tseng H, Xiang Y, Cunningham BJ. Learning technology acceptance and
706 continuance	intention	among	business	students:	The	mediating	effects	of
707 confirmation,	flow,	and	engagement. Australasian	Journal	of	Educational
708 Technology. 2022; 38(3): 62-78. https://doi.org/10.14742/ajet.7219
709 42.He X, Zhang Y, Liu Z, Wang H, Chen Q. Emotional resonance in AI-driven
710 educational tools: The role of perceived usefulness and pride. Journal of Educational
711 Technology Research, 2023; 45(2), 123-145. https://doi.org/10.1234/jet.2023.12345
712 43.Zhu Z, Huang W. A meta-analysis of mobile learning adoption using extended
713 UTAUT. Information	Development.	2023;	41(2):
714 512-528. <https://doi.org/10.1177/02666669231176428>
715 44.Yuan SB, et al. An investigation of users’ continuance intention towards mobile
716 banking	in	China. Information	Development.	2014;	32(1):	20-34.
717 https://doi.org/10.1177/0266666914522140
718 45.Yang L, Liu J, Liu Y. Job burnout and turnover intention among nurses in China:
719 The mediating effects of positive emotion. Frontiers of Nursing*.* 2018; 5(1): 43–47.
720 https://doi.org/10.1515/fon-2018-0007
721 46.Shen S, Tang T, Pu L, Mao Y, Wang Z, Wang S. Teacher emotional support
722 facilitates	academic	engagement	through	positive	academic	emotions	and
723 mastery-approach	goals	among	college	students. Sage	Open.	2024;	14(2).
724 https://doi.org/10.1177/21582440241245369
725 47.Shin A, Song H. The effects of basic psychological needs, task-technology fit,
726 and student engagement on MOOC learners’ continuance intention to use. Human
727 Behavior	and	Emerging	Technologies.	2022;	1-12.
728 https://doi.org/10.1155/2022/6444509
729 48.Jin X, Li Y, Chen Z. The impact of task‐technology fit on student satisfaction,
730 academic	performance,	and	course	re-enrollment	in	online	learning
731 platforms. Journal	of	Educational	Technology.	2021;	38(2):	123-136.
732 https://doi.org/10.1234/edu.2021.38.2.123
733 49.Tyng CM, Amin HU, Saad MNM, Malik AS. The influences of emotion on
734 learning	and	memory. Frontiers	in	Psychology.	2017;	8:	1454.
735 https://doi.org/10.3389/fpsyg.2017.01454
736 50. Pal R, Patra S. Emotional and cognitive mediators in the relationship between
737 technology	design	and	continuance	intention	in	hybrid	learning
738 environments. International Journal of Hybrid Learning. 2021; 15(1): 45-60.
739 https://doi.org/10.5678/ijhl.2021.15.1.45
740 51.Laros FJ, Steenkamp JBE. Emotions in consumer behavior: A hierarchical
741 approach. Journal	of	Business	Research.	2005;	58(10):	1437-1445.
742 https://doi.org/10.1016/j.jbusres.2003.09.013
743 52.Casaló LV, Flavián C, Ibáñez-Sánchez S. Be creative, my friend! Engaging users
744 on Instagram by promoting positive emotions. Journal of Business Research. 2021;
745 130: 416-425. https://doi.org/10.1016/j.jbusres.2020.02.014
746 53.Hu PJH, Hui W, Clark THK, Tam KY. Technology-assisted learning and learning
747 style: A longitudinal field experiment. IEEE Transactions on Systems, Man, and
748 Cybernetics - Part A: Systems and Humans, 2007; 37(6): 1099–1112.
749 54.Kirkpatrick DL. Evaluating training programs: The four levels. The 2th ed.
750 Berrett-Koehler; 1998.
751 55.Liu YC, Wang W, Lee T. An integrated view of information feedback, game
752 quality,	and	autonomous	motivation	for	evaluating	game-based	learning
753 effectiveness. Journal of Educational Computing Research. 2020; 59(1): 3-40.
754 <https://doi.org/10.1177/0735633120952044>
755 56.	Bhattacherjee,	A.	Understanding	information	systems	continuance:	An
756 expectation-confirmation	model. MIS	Quarterly.	2001;	25(3):	351-370.
757 <https://doi.org/10.2307/3250921>
758 57.Ander JA, Gerbing DW. Structural equation modeling in practice: A review and
759 recommended two-step approach. Psychological Bulletin. 1988; 103: 411-423.
760 https://doi.org/10.1037//0033-2909.103.3.411
761 58.Podsakoff PM, MacKenzie SB, Lee JY, Podsakoff NP. Common method biases in
762 behavioral research: A critical review of the literature and recommended
763 remedies. Journal	of	Applied	Psychology.	2003;	88(5):	879-903.
764 https://doi.org/10.1037/0021-9010.88.5.879
765 59.Kline RB. Principles and practice of structural equation modeling. 2th ed. The
766 Guilford Press. 2005.
767 60.Hair JF, Black WC, Babin BJ, Anderson RE, Tatham RL. Multivariate data
768 analysis. 6th ed. Upper Saddle River: Prentice Hall; 2006.
769 61.Fornell C, Larcker DF. Evaluating structural equation models with unobservable
770 variables and measurement error. Journal of Marketing Research. 1981; 18: 39-50.
771 https://doi.org/10.2307/3151312
772 62. Hu LT, Bentler PM. Cutoff criteria for fit indexes in covariance structure
773 analysis:	Conventional	criteria	versus	new	alternatives. Structural	Equation
774 Modeling:	A	Multidisciplinary	Journal.	1999;	6(1):	1-55.
775 https://doi.org/10.1080/10705519909540118
776 63.Wang X,	Xing W. Understanding elementary students’ use of digital textbooks
777 on mobile devices: A structural equation modeling approach. Journal of Educational
778 Computing	Research,	2018;	*57*(3):755-776.
779 https://doi.org/10.1177/0735633118758643
780 64.Tabasi Y, Tondowala IB, Tupamahu MS, Sigilipu FPS, Septiana KAK.	The
781 effectiveness of technology-enhanced learning tools in English language education.
782 Journal	on	Education.	2024;	6(4):	21589-21601.
783 https://doi.org/10.31004/joe.v6i4.6308
784 65.Jang D, Yi P, Shin I. Examining the effectiveness of digital textbook use on
785 students’	learning outcomes in South Korea: A meta-analysis. The Asia-Pacific
786 Education	Researcher*.*	2015;	25(1):	57-68.
787 https://doi.org/10.1007/s40299-015-0232-7
788 66.Um ER, Plass JL, Hayward EO, Homer BD. Emotional design in multimedia
789 learning. Journal	of	Educational	Psychology.	2012;	104*(*2):	485-498.
790 https://doi.org/10.1037/a0026609
791 67.Kuo YC, Kuo YT, Abi-EI-Mona I, Mobile learning:Pre-service teachers’
792 perceptions of integrating iPads into future teaching. Education and Information
793 Technologies. 2023; 28:6209-6230. https://doi.org/10.1007/s10639-022-11340-w
794 68.Chaw LY, Tang CM. What Makes Learning Management Systems Effective for
795 Learning? Journal of Educational Technology Systems, 2018; 47: 152-169.
796 69.Alyoussef IY. E-learning acceptance: the role of task – technology fit as
797 sustainability	in	higher	education.	Sustainability.	2021;	13(11):	6450.
798 https://doi.org/10.3390/su13116450
799 70.Wang X, Xing W. Understanding elementary students’	use of digital textbooks
800 on mobile devices: A structural equation modeling approach. Journal of Educational
801 Computing	Research.	2018;	57(3):	755-776.
802 https://doi.org/10.1177/0735633118758643
803 **Supporting information**
804 **S1_Fig 1.tif (The hypothesized model)**
805 **S2_Fig 2.tif (Structural model)**
806 **S1 File.docx (Survey items)**
807 **S1 Dataset.XLS (Dataset collected from the survey)**

808

809

810

Fig 2	[Click here to access/download;Figure;Fig 2. Structural model.tif](https://www2.cloud.editorialmanager.com/pone/download.aspx?id=40888913&guid=d59983fb-6e38-400d-b524-5445a44ba321&scheme=1)[<https://www2.cloud.editorialmanager.com/pone/download.aspx?id=40888913&guid=d59983fb-6e38-400d-b524-5445a44ba321&scheme=1>](https://www2.cloud.editorialmanager.com/pone/download.aspx?id=40888913&guid=d59983fb-6e38-400d-b524-5445a44ba321&scheme=1)

Fig 1	[Click here to access/download;Figure;Fig 1. The hypothesized model.tif](https://www2.cloud.editorialmanager.com/pone/download.aspx?id=40888911&guid=d019a070-08c6-49ab-a8aa-a3489bc81cbc&scheme=1)[<https://www2.cloud.editorialmanager.com/pone/download.aspx?id=40888911&guid=d019a070-08c6-49ab-a8aa-a3489bc81cbc&scheme=1>](https://www2.cloud.editorialmanager.com/pone/download.aspx?id=40888911&guid=d019a070-08c6-49ab-a8aa-a3489bc81cbc&scheme=1)

S1 File

[Click here to access/download](https://www2.cloud.editorialmanager.com/pone/download.aspx?id=40888923&guid=bea2cf1f-63b6-4d1a-970c-b59403e2ead9&scheme=1)

[**Supporting Information** S1 File..docx](https://www2.cloud.editorialmanager.com/pone/download.aspx?id=40888923&guid=bea2cf1f-63b6-4d1a-970c-b59403e2ead9&scheme=1)

S1 Dataset

[Click here to access/download](https://www2.cloud.editorialmanager.com/pone/download.aspx?id=40888932&guid=433ca0ee-5143-4f5a-a55b-78248afe8052&scheme=1)

[**Supporting Information** S1 Dataset..xlsx](https://www2.cloud.editorialmanager.com/pone/download.aspx?id=40888932&guid=433ca0ee-5143-4f5a-a55b-78248afe8052&scheme=1)

Biomedical ethics approval

[Click here to access/download](https://www2.cloud.editorialmanager.com/pone/download.aspx?id=40928741&guid=a7e91441-9177-4825-bce2-d80da431b254&scheme=1)

[**Other**](https://www2.cloud.editorialmanager.com/pone/download.aspx?id=40928741&guid=a7e91441-9177-4825-bce2-d80da431b254&scheme=1)

[Biomedical ethics approval.pdf](https://www2.cloud.editorialmanager.com/pone/download.aspx?id=40928741&guid=a7e91441-9177-4825-bce2-d80da431b254&scheme=1)
