# Supplementary material for: Task–technology fit and digital textbook usage outcomes: The mediating role of positive emotion within an S–O–R framework
Source: PLoS One. 2026 Mar 10;21(3):e0344382. doi: 10.1371/journal.pone.0344382 (PMC12974882; doi:10.1371/journal.pone.0344382)
Supplement: Attachment — Submitted filename: Response to Reviewers.docx [file pone.0344382.s006.docx]

### **Response to Reviewers**

Dear Editor and Reviewers,

We sincerely thank you for the time and effort devoted to reviewing our manuscript entitled “Task-Technology Fit Drives Digital Textbook Continuance: Serial Mediation of Positive Emotion and Learning Effectiveness.” We truly appreciate the insightful comments and constructive suggestions, which have helped us improve the clarity, coherence, and overall quality of the paper.

All revisions have been incorporated into the revised manuscript, with changes highlighted using the Track Changes. We have also submitted a clean version of the revised manuscript and the present response document detailing how each comment has been addressed.

After carefully considering all feedback, we provide a detailed, point-by-point response below. Since the reviewers’ comments were provided in the form of in-text annotations, we have included the original text to make the reviewers’ comments and our corresponding revisions clearer. Each reviewer’s comment is presented in italic font, followed by our response in plain text.

Comment 1: *Is there any relation with SR theory by Skinner or Pavlov? Just a question.*

Response: The S-O-R (Stimulus-Organism-Response) model is indeed conceptually related to the classical SR (Stimulus-Response) framework proposed by Pavlov and further developed by Skinner, in that both focus on the impact of external stimuli on behavior. However, while SR theory emphasizes a direct link between stimulus and response, S-O-R extends this framework by incorporating the internal state of the organism (O), such as cognitive, emotional, or motivational processes, as a mediating factor. In our study, this extension is particularly relevant because we aim to examine how perceived task-technology fit (TTF) affects students’ continuance intention (COI) via sequential internal processes—positive emotion (POE) and learning effectiveness (LEE). Therefore, S-O-R provides a more suitable theoretical basis than the traditional SR framework, as it allows us to account for the mediating roles of affective and cognitive responses in the use of digital textbooks.

Comment 2: *Have the authors personally noted that digital books adversely affect the ultimate learning?*

Response: We sincerely appreciate the reviewer’s comment. While in practice we have occasionally observed that digital textbooks may have certain adverse effects on students’ learning, such as distractions or over-reliance on digital features, this study focused on the positive mechanisms through which perceived task-technology fit (TTF) influences continuance intention (COI), via positive emotion (POE) and learning effectiveness (LEE). Our data indicate that, within the context of this study, the overall effect of TTF on learning effectiveness and continuance intention is positive. Acknowledging potential negative effects highlights areas for future research but does not change the main findings regarding the sequential mediating roles of POE and LEE.

Comment 3: *Not only EFL but all landscapes/environments*

Original: In recent years, there has been a rapid increase in the use of digital textbooks in English as a Foreign Language (EFL) education.

Response: We greatly appreciate this insightful comment. Indeed, digital textbooks have been used not only in the field of EFL learning and teaching, but other fields. Therefore, we revised the original sentence as follows:

In recent years, there has been a rapid increase in the use of digital textbooks across various educational contexts.（Introduction, page 1, line 21-22）

Comment 4: *You mean barriers/obstacles or learning difficulties?*

Original: However, use of digital textbooks by many EFL learners halts after some time, which reduces their long-term educational value [3].

Response: We thank the reviewer for this insightful comment, which has helped us improve the manuscript. By “halts,” we meant that many learners gradually discontinue or reduce their use of digital textbooks over time, rather than encountering specific barriers or obstacles. We will revise the manuscript to clarify this point and use more precise phrasing, such as “decreases”, to avoid ambiguity. Considering the coherence with the topic of this paper, in accordance with the Comment 3, we have revised the sentence as follows:

However, despite these benefits, in some contexts including English as a Foreign Language (EFL) learning, learners’ use of digital textbooks often decreases over time, limiting their long-term educational value [3]. (Introduction, page 1, line 26-28)

Comment 5: *And affordances too*

Original: According to past studies [5], TTF affects whether users find a technology useful, are satisfied with one, and continue using one.

Response: We thank the reviewer for this insightful suggestion. We agree that affordances are conceptually related to TTF in shaping users’ perceptions of technology use. We have therefore revised the sentence to include affordances, highlighting their relevance to perceived usefulness and continued usage. The revised sentence is as follows:

According to past studies [5], TTF affects whether users find a technology and its affordances useful, are satisfied with it, and continue using it. (Introduction, page 2, line 34-35)

Comment 6: *Is it not a big claim?*

Original: Despite this, researchers do not have a good understanding of the connection’s thinking and emotional processes.

Response: We sincerely appreciate the reviewer’s valuable and constructive comment. We agree that the original statement may be too strong. A more precise way to express this is that the connection between task-technology fit (TTF) and learners’ cognitive and emotional processes has been relatively underexplored, and our study aims to provide further insight into these mechanisms. The revised sentence is as follows:

Despite this, the connection between TTF and learners’ cognitive and emotional processes has been relatively underexplored. (Introduction, page 2, line 39-40)

Comment 7: *Digital support will be better*

Original: The good fit between the aid and the task pursued by EFL learners is a primary reason for continued use, which therefore could occur through either emotional or mental processes.

Response: We appreciate the reviewer’s comment. The sentence has been revised accordingly as follows:

The good fit between the digital support and the task pursued by EFL learners is a primary reason for continued use, which therefore could occur through either emotional or mental processes. (Introduction, page 2, line 44)

Comment 8: *Take care: curiosity is neither a variable nor a stimulant in many cases*

Original: The recent studies state positive emotions such as enjoyment, curiosity, and satisfaction are vital to learning with technology.

Response: We appreciate the reviewer’s insightful observation. We agree that curiosity may not always function as a stable affective variable or stimulus in technology-enhanced learning contexts. To address this, we have replaced “curiosity” with “pride”, which is recognized as a key positive emotion influencing learners’ motivation and engagement in digital language learning. Recent empirical studies (e.g., Wang et al., 2024; Zou et al., 2025) have confirmed that pride, together with enjoyment, functions as an important positive emotional factor that promotes self-regulated learning, motivation, and persistence in L2 learning contexts. This aligns well with our study’s focus on affective mechanisms underlying technology-mediated learning continuance. The revised sentence is as follows:

Recent studies state that positive emotions such as enjoyment, pride, and satisfaction are vital to learning with technology. (Introduction, page 2, line 47)

Comment 9: *have been*

Original: Consequently, three research questions that follow should be addressed:

Response: We appreciate the reviewer’s suggestion. However, this sentence appears in the introduction to indicate the research purpose rather than to describe completed work. Therefore, we retained “should be addressed” to emphasize that these questions need to be explored in the present study.

Comment 10: *Why not ‘to what extent’?*

Original: 1.How does TTF influence EFL learners’ COI to use digital textbook?

Response: We appreciate the reviewer’s thoughtful observation. The purpose of research question 1 is to explore how task-technology fit (TTF) influences learners’ continuance intention (COI), focusing on the mechanisms and pathways of this influence rather than the magnitude or degree of the effect. Since our study aims to examine the mediating processes (i.e., positive emotion and learning effectiveness) rather than quantify the extent of impact, we believe that using “How” is conceptually more appropriate in this context.

Comment 11: *Why not educational psychology?*

Original: Rooted in environmental psychology, the S-O-R mechanism establishes that diverse environment attributes function as stimulus (S) shaping individuals’ affective-cognitive state (O), thereby eliciting behavioral responses (R).

Response: We sincerely thank the reviewer for this thoughtful question. The expression “environmental psychology” is retained to reflect the theoretical origin of the S-O-R framework, which was first introduced by Mehrabian and Russell (1974) to explain how environmental stimuli affect individuals’ internal states and behaviors. In the subsequent sentences of this section, we have already discussed its extensive applications in digital learning and behavioral studies, which implicitly cover the field of educational psychology. Therefore, we kept the term “environmental psychology” to indicate the model’s origin while acknowledging its broad applicability across educational and technological contexts.

Comment 12: *Is there a role of students’ preparedness in the process?*

Original: The link between TTF and COI is based on a core idea of TTF theory: when a technology matches the user’ s task requirement, it is perceived as more useful and efficient, thereby fostering continued usage [4]. Studies in educational technology show that when TTF is high, it reduces mental effort and reinforces the user’s perception of the tool usefulness. These two outcomes are important in predicting whether learners will continue using digital learning tools, especially those based in the cloud [24]. Wang et al. [25] also pointed out that TTF is especially important in learning settings that involve repeated practice, like language learning. Based on the previous research, we put forward the the first hypothesis:

H1: TTF has a direct positive impact on EFL learners’ COI to use digital textbooks.

Response: We sincerely appreciate the reviewer’ s insightful comment. We understand that the reviewer is suggesting that learners’ preparedness, such as their prior digital learning experience, self-regulation ability, or readiness to adopt new tools, may influence how well task-technology fit (TTF) affects their continuance intention (COI). We agree that students’ preparedness can moderate the relationship between TTF and COI, since learners with higher preparedness may perceive the technology as more useful and are more likely to continue using it. However, in the current study, we focused primarily on the direct effect of TTF on COI, mediated by constructs within the S-O-R framework. We therefore did not include preparedness as a moderating variable in the model to maintain conceptual parsimony. To acknowledge the reviewer’s point, we have added additional sentences to explicitly highlight this point in the revised manuscript (Limitations and future directions, page 22, line 551-554).

Comment 13: *Over enjoyment becomes funny and they don’ t take it seriously, just an idea.*

Response: We appreciate the reviewer’s insightful observation. We agree that while enjoyment is generally a positive factor that promotes learning engagement and continuance intention, too much enjoyment may reduce students’ focus or seriousness. This valuable point suggests that future research could explore how different levels of enjoyment influence learning outcomes.

Comment 14: *Any connection with affective domain related to Bloom’ s taxonomy?*

Comment 15: *Is it somewhat connected to Bandura’ s theory?*

Original: Task-technology fit, learning effectiveness and continuance intention

Previous research confirmed the critical influence of task-technology congruence on LEE and COI development [28]. Furthermore, Shin and Song [47] demonstrated that learning engagement, closely tied to learning effectiveness, mediates the relationship between TTF and students’ continued intention to use educational technologies. Their study demonstrated that perceived congruence between learning tools and academic tasks promotes learning efficacy, thereby reinforcing continuance intention toward digital textbook learning. Therefore, we proposed:

H8: Learning effectiveness mediates the relationship between task-technology fit and continuance intention in the context of digital textbooks.

Response to comment 14: We thank the reviewer for this insightful comment. While Bloom’s affective domain relates to learners’ emotions and motivation, in our study positive emotion is treated as an independent antecedent influencing learning effectiveness, rather than being incorporated within learning effectiveness itself. Therefore, there is an indirect conceptual connection to the affective domain, but positive emotion and learning effectiveness are modeled as distinct constructs in accordance with the S-O-R framework. We hope this explanation clarifies the rationale behind H8 and addresses the reviewer’s comment, without requiring any modification to the original manuscript.

Response to comment 15: We would like to clarify that the current construct of learning effectiveness differs from Bandura’s concept of self-efficacy. While self-efficacy refers to learners’ belief in their ability to perform specific tasks, learning effectiveness in our model focuses on observable behavioral and cognitive outcomes of digital textbook use. To avoid confusion, we have revised the wording in the manuscript accordingly as follows:

Their study demonstrated that perceived congruence between learning tools and academic tasks promotes learning effectiveness, thereby reinforcing continuance intention toward digital textbook learning. (Theoretical background and hypotheses development, page 7, line 194)

Comment 16: *Delete one*

Original: Task-technology fit, positive emotion, positive emotion and continuance intention

Response: The repeated term “positive emotion” has been removed as suggested (Theoretical background and hypotheses development, page 8, line 198-199).

Comment 17: *Does it affect intrinsic motivation too?*

Original: TTF regarded as external stimulus in online learning platforms enhanced students’ satisfaction, which improved their academic performance as cognitive organism, ultimately increasing their likelihood of course re-enrollment as a response [48].

Response: While our study focused on cognitive and behavioral outcomes, we acknowledge that task-technology fit may also influence learners’ intrinsic motivation. Although this was not measured in the current study, we recognize this as an important avenue for future research.

Comment 18: *May be considered or conceived*

Original: Learning effectiveness is operationally perceived as the measurable degree to which learners successfully internalize targeted knowledge, develop competencies, and master domain-specific skills [53].

Response: We thank the reviewer for the helpful suggestion. Following your advice, we have replaced “perceived” with “considered” to better reflect the operational definition of learning effectiveness from the researcher’s perspective. The revised sentence is as follows:

Learning effectiveness is operationally considered as the measurable degree to which learners successfully internalize targeted knowledge, develop competencies, and master domain-specific skills [53]. (Method, page 9, line 247)

Comment 19: *exceeds*

Original: Discriminant validity was evaluated using the criterion [61], which requires that the square root of each construct’s AVE exceed its bivariate correlations with all other constructs.

Response: Following the suggestion, we have corrected the verb from “exceed” to “exceeds” in the sentence. The revised sentence is as follows:

Discriminant validity was evaluated using the criterion [61], which requires that the square root of each construct’s AVE exceeds its bivariate correlations with all other constructs. (Results, page 14, line 346)

Comment 20: *grammar*

Response: The typographical error in the column heading “Critieria for Judgement” (Table 4) has been corrected to “Criteria for Judgement” in the revised manuscript (Results, page 15).

Comment 21: *Continue to use*

Original: The study aims to dig out the relationships among task-technology fit, positive emotion, learning effectiveness, and continuance intention to use digital textbook, with a particular focus on EFL learners.

Response: We appreciate the reviewer’s suggestion. However, we would like to clarify that the term “continuance intention” already denotes the intention to continue using a technology. Therefore, adding “continue to use” after it would lead to redundancy. Hence, we have retained the original expression for precision and consistency with existing literature.

Comment 22: *Is it voluntary, optional or mandatory or simultaneous with printed matter?*

Original: The high predictive power of POE on COI (H4) and LEE (H5) suggests that students continue to use the digital textbook when they see its effectiveness.

Response: We appreciate the reviewer’s insightful question about the contextual nature of digital textbook usage. In our research setting, printed textbooks are mandatory course materials prescribed by the university, while the digital textbook serves as a complementary learning resource within the same course. Students are encouraged but not required to use the digital textbook. Therefore, their engagement and continuance intention toward the digital version are voluntary and self-directed. This means that the construct of continuance intention in our study reflects learners’ intrinsic motivation and perceived usefulness, rather than compliance with institutional mandates. We have clarified this contextual information in the revised manuscript as follows:

The high predictive power of POE on COI (H4) and LEE (H5) suggests that students tend to continue engaging with the digital textbook as a supplementary learning resource when they perceive it as effective and beneficial to their learning. (Discussion, page 18, line 427-430)

Comment 23: *eventually*

Original: TTF increases students’ positive emotions, which then improve their learning effectiveness.

Response: Following the suggestion, we have corrected the adverb from “then” to “eventually” in the sentence. The revised sentence is as follows:

TTF increases students’ positive emotions, which eventually improve their learning effectiveness. (Discussion, page 19, line 466)

Comment 24: *How does this study contribute to the understanding of digital learning of English in China?*

Original: These findings support earlier work by Zhao and Bação [3], who identified TTF affects both emotional and learning outcomes, and by Alyoussef [69], who found that matching tasks with technology supports digital learning through emotional balance and effective study.

Response: We appreciate this constructive comment. To clarify the contextual contribution, we have added a sentence to emphasize the relevance of the findings to digital English learning in China. The added sentence is as follows:

In particular, they enrich our understanding of how task-technology alignment fosters effective and emotionally engaging digital English learning in the Chinese EFL context. (Discussion, page 19, line 474-476)

Comment 25: *offer*

Original: These results give new ideas for future research and better ways to design digital learning tools that improve student involvement and learning success.

Response: We have corrected the verb from “give” to “offer” as follows:

These results offer new ideas for future research and better ways to design digital learning tools that improve student involvement and learning success. (Theoretical and pedagogical implications, page 20, line 490)

Comment 26: *creates*

Comment 27: *Punctuation issue*

Original: This comprehensive approach offers a novel insight into the mechanisms through which psychological stimulus such as perceived TTF affects organisms like POE and perception of LEE, which subsequently promote continuance usage intention.

Response to comment 26: We have corrected the verb from “offers” to “creates” (page 20, line 492).

Response to comment 27: We thank the reviewer for pointing out the punctuation and clarity issue. We have revised the sentence for improved readability and precision. The revised sentence is as follows:

This comprehensive approach creates a novel insight into the mechanisms through which external stimuli, such as perceived task-technology fit (TTF), influence organismic factors like positive emotion (POE) and the perception of learning effectiveness (LEE), which subsequently promote continuance usage intention. (Theoretical and pedagogical implications, page 20, line 492-496)

Comment 28: *And pedagogues in*

Original: From a practical standpoint, the findings from this research hold substantial implications for digital textbook content compilers and technology designers, as well as policy-makers in the realm of EFL education.

Response: We thank the reviewer for this helpful suggestion. We have revised the sentence to include pedagogues as additional stakeholders. This modification ensures that both educational practitioners and decision-makers are recognized as relevant beneficiaries of the research. The revised sentence is as follows:

From a practical standpoint, the findings from this research hold substantial implications for digital textbook content compilers, technology designers, pedagogues, and policy-makers in the realm of EFL education. (Theoretical and pedagogical implications, page 20, line 499-501)

We hope that the revisions meet the standards for publication in PLOS ONE and look forward to your further consideration.

Sincerely,
Ning Mi
On behalf of co-author

Department of College English Teaching
Qufu Normal University, Qufu, Shandong, China
Email: sdmining@qfnu.edu.cn
