# Supplementary material for: Task–technology fit and digital textbook usage outcomes: The mediating role of positive emotion within an S–O–R framework
Source: PLoS One. 2026 Mar 10;21(3):e0344382. doi: 10.1371/journal.pone.0344382 (PMC12974882; doi:10.1371/journal.pone.0344382)
Supplement: Attachment — Submitted filename: PONE-D-25-33273R1..docx [file pone.0344382.s005.docx]

Task-technology fit drives digital textbooks continuance: Serial mediation of positive emotion and learning effectiveness

1. The article showed clear problem framing. The decline in COI of digital textbooks in EFL contexts is well established. The importance of post-adoption behavior is clearly justified. This aligns well with PLOS ONE’s preference for clearly motivated applied research.
2. However, the articulation of contribution is somewhat weak. You repeatedly state that “few studies have examined emotions and cognition jointly” but this claim is too general. Not sufficiently bounded to EFL digital textbooks. Not explicitly contrasted with what existing studies already do. PLOS ONE requires a clearly stated incremental contribution even if modest, it must be explicit and concrete. You must clearly state what is new, in terms of contextual (EFL digital textbooks specifically), structural (serial mediation vs parallel mediation), and/ or theoretical integration (explicit S-O-R operationalization of TTF).
3. There are overlapping hypotheses (H7–H9). H7, H8, and H9 are conceptually overlapping:

- H7: POE mediates TTF → COI
- H8: LEE mediates TTF → COI
- H9: POE → LEE serial mediation

For reviewers, this can appear as hypothesis inflation. This is not incorrect, but it must be justified explicitly as, testing partial vs full mediation and/or comparing parallel vs serial mechanisms.

4. Issues related to language precision and academic tone must be addressed. This includes minor grammatical issues (e.g., “learner’ s”), occasional informal phrasing (“great understanding”), inconsistent pluralization (“digital textbook” vs “digital textbooks”).
5. In Methods section, the description of the Output-Oriented Approach and philosophical elements (“integration of culture and Taoism”) is overly descriptive and pedagogical, not methodological. Prioritize method relevance, not curriculum exposition.
6. You stated, “All scales underwent rigorous validation to ensure construct reliability and validity prior to deployment.” This is misleading. Validation occurred during analysis, not prior to deployment. This requires revision. Rephrase to: “All scales were adapted from validated instruments and subsequently assessed for reliability and validity in the present study.” This is essential to avoid methodological misrepresentation.
7. You relied only on Harman’s single-factor CFA, which can be considered insufficient alone. Do not claim CMV is “not a problem.” Add procedural remedies already implied, anonymity, voluntary participation, and separation of constructs.
8. The high correlation between COI and LEE (0.832) may imply multicollinearity which is vital in SEM. Present VIF results and address possible concern.
9. It was stated that, “The square root of AVE for LEE fell slightly below its correlation…” This technically violates Fornell–Larcker. What is your solution on this?
10. Discussion is repetitive and descriptive, with limited abstraction. Condense repetitive confirmations and elevate synthesis. Focus more on mechanisms, less on restating coefficients. Emphasize why LEE dominates COI.
11. The Pedagogical & Policy Implication section overreaches empirically, especially, “…emotion recognition interfaces that dynamically adjust content difficulty…” Your study did not measure emotion recognition technology. Reframe as speculative future directions, not implications.
