# Supplementary material for: Task–technology fit and digital textbook usage outcomes: The mediating role of positive emotion within an S–O–R framework
Source: PLoS One. 2026 Mar 10;21(3):e0344382. doi: 10.1371/journal.pone.0344382 (PMC12974882; doi:10.1371/journal.pone.0344382)
Supplement: Attachment — Submitted filename: Response to Reviewers_Revised_v2.docx [file pone.0344382.s007.docx]

### **General Response:**

We sincerely thank the Editor and reviewers for their constructive and detailed feedback. We have carefully revised the manuscript in response to all comments and made additional improvements to enhance clarity, coherence, and the theoretical framing of the study.

Although the reviewers did not explicitly request a change of title, the title has been updated to “Task–Technology Fit and Digital Textbook Usage Outcomes: The Mediating Role of Positive Emotion within an S–O–R Framework” to better reflect the guiding theoretical framework and the revised conceptual model, in which the dependent construct is specified as a second-order factor.

It should be clarified that the empirical foundation of this study remains unchanged despite the title change: the questionnaire, sample, and dataset are identical to those in the original submission, with ethical approval [2025-087] still valid. The revision is confined to theoretical framing, model specification, and interpretive approach.

Below, we provide a point-by-point response to each comment.

**Review Comments to the Author**

**Reviewer #2:**

**Comment 1**—Language and flow: Minor grammatical and typographical errors are present. A thorough proofread is recommended. Examples include:
• “student’ s” in line 12 (page 1).
• “the the” in H1 description.
• Lines 170-171 (page 6) and 197-198 (page 7) are repetitive.
• “LOE” instead of “LEE” in line 335 (page 13).
• Factor loading for LEE2 “0.972” in Table 2 instead of “0.87” in Figure 2.
• “TTF (AVE = 0.717” in line 340 (page 13) instead of “0.715” in Table 2.
• “LEL” in Table 5 path label.

**Response:**
We thank the reviewer for the meticulous reading of our manuscript and for identifying technical and linguistic inconsistencies. In response, the manuscript has undergone comprehensive restructuring and revision to address both the major theoretical updates and the specific issues noted.

1.Language and typos: All typographical errors and repetitive expressions (e.g., “the the,” “student’ s”) have been corrected or removed. The manuscript has been professionally proofread to ensure grammatical accuracy and improved readability.

2.Terminology and data consistency: With the revised model, in which LEE and COI are integrated into the second-order construct DTUO, all tables, figures, and statistical values have been recalculated and verified. Inconsistencies in AVE values, factor loadings, and construct labels (e.g., “LEE/LOE/LEL”) have been corrected to ensure alignment between text and tables.

3.Accuracy verification: All reported data points were cross-checked against the updated structural equation model outputs to ensure correctness.

These revisions have enhanced the rigor, clarity, and coherence of the manuscript.

**Comment 2—Methods section: Justify the sample size and the sampling technique used, and discuss potential sources of bias.**

**Response:**

### We thank the reviewer for this suggestion. In the revised manuscript, the Participants section has been expanded to provide justification for the sample size, clarify the sampling technique, and address potential biases.

### 1.Sample Size Justification: The sample of 259 was justified using two benchmarks: the absolute minimum of 200 for stable SEM estimates (Kline, 2016) and the case-to-indicator ratio. With 17 indicators, our ratio of 15.2:1 exceeds the recommended 5:1–10:1 range (Hair et al., 2019; Bentler & Chou, 1987), ensuring adequate statistical power.

### 2.Sampling Technique: Convenience sampling was used because participants were active, representative users of digital textbooks in their required English courses. This approach ensured that the data reflected direct experience with the technology, essential for assessing task–technology fit, positive emotion, and usage outcomes.

### 3.Potential Biases: A paragraph was added discussing potential biases, including limits of generalizability from a single-university sample, gender distribution, and self-report bias. Procedural remedies, such as anonymity and emphasizing that there were no right or wrong answers, were also described.

### Revised Section: See Participants (Page 9, Lines 220–249).

### **Comment 3—Methods section: Provide more detail on scale adaptation (e.g., expert review, pilot testing) and include a table summarizing all constructs, items, and sources in the main text or supplement.**

**Response:**

### We thank the reviewer for this constructive suggestion. To enhance rigor and transparency, the Instrument section has been revised as follows:

### 1.Scale Adaptation: We elaborated on the adaptation process following standard cross-cultural procedures (Brislin, 1970), including forward–backward translation, expert review for content validity, and a pilot test (N = 30) to refine item wording for clarity.

### 2.Summary Table: A new table (Table 1.) has been added in the main text summarizing all constructs, dimensions, items, sources.

### Revised Section: See Instrument (Pages 10–12, Lines 270–314).

### **Comment 4—Results section: Explore more robust methods to test for CMV or acknowledge that the used test, while common, is a basic one, and that the use of self-report data from a single source is a study limitation.**

**Response:**

We thank the reviewer for highlighting the need for more rigorous assessment of common method variance (CMV). We agree that the single-factor test, while common, is a preliminary check and that reliance on self-report data from a single source is a limitation.

In the revised manuscript, we implemented a two-step procedure to address CMV in our updated model (where LEE and COI are integrated into the second-order DTUO construct):

1.Preliminary Check: A single-factor confirmatory factor analysis (CFA) was conducted. Poor model fit ($\chi^{2}/df$=7.047, AGFI = 0.535, GFI = 0.638, and RMSEA = 0.153) indicated that a single factor does not account for the majority of variance.

2.Robustness Assessment: We applied the Unmeasured Latent Method Construct (ULMC) approach [Liang et al., 2007; Podsakoff et al., 2003]. Differences in standardized factor loadings between models with and without the common latent factor ranged from 0.000 to 0.073, well below the conservative threshold of 0.200 [Cohen, 1988], indicating that CMV does not substantially bias the observed relationships.

These results are reported in the updated Common Method Variance section (see Pages 13–14, lines 347–371). We also explicitly acknowledge the limitation of single-source self-report data in the revised Limitations section (see Pages 27–28, lines 705–744).

### **Comment 5—Descriptive statistics and correlation analysis section: The authors’ claim about Kline's thresholds is inaccurate. Correcting this statement in a final revision would improve the manuscript's scholarly precision. Authors are encouraged to support the results with a normality test.**

### **Response:**

### We thank the reviewer for pointing out the inaccuracy regarding Kline’s thresholds. In the revised manuscript, we have corrected the criteria, defining severe non-normality as absolute skewness > 3 and kurtosis > 10, in line with Kline’s recommendations.

### Additionally, following the reviewer’s suggestion, we conducted a Shapiro–Wilk normality test, and the results have been incorporated. Given the test’s sensitivity to sample size, skewness and kurtosis were also examined as complementary indicators of distributional form. These revisions provide a more precise and theoretically grounded assessment of the data’s distributional properties.

### The updated content can be found in the Descriptive Statistics and Correlation Analysis section (Pages 14–15, lines 372–386).

### **Comment 6—Structure model analysis section: Discuss the independence of observations, multicollinearity, linearity, and statistical stability of the results.**

### Response: We thank the reviewer for emphasizing the importance of verifying the statistical assumptions underlying the structural model. Following the revision of the research model—where LEE and COI were specified as dimensions of the higher-order construct DUTO—we conducted diagnostic checks and added a new subsection, Assumptions of Multivariate Analysis, at the beginning of the Structural Model Analysis section. The results are summarized below:

### 1.Independence of observations: Participants completed the questionnaire individually, ensuring independence. Residual independence was also examined using the Durbin–Watson statistic for the regression of TTF and POE on DUTO (value = 1.810), which falls within the acceptable range (1.5–2.5), indicating minimal autocorrelation.

### 2.Multicollinearity: Variance inflation factors (VIFs) for TTF and POE were 1.598, well below the conservative threshold of 3.0 (Hair et al., 2019), suggesting that multicollinearity is unlikely to substantially bias the estimates.

### 3.Linearity: The linearity of relationships was evaluated through the specified SEM structure and overall model fit. Acceptable fit indices (χ²/df < 3, CFI > 0.90, RMSEA < 0.08) indicate consistency between the linear model and the observed data.

### 4.Statistical stability: Stability of parameter estimates was assessed using 5,000-resample bootstrapping to generate 95% confidence intervals, and a latent method factor test confirmed that substantive loadings remained robust after controlling for potential method effects.

### These diagnostic checks collectively support the robustness and reliability of the revised structural model.

### (See Page Pages 17–18, lines 438–456 in the revised manuscript for details.)

**Comment 7—Limitations: Acknowledge more explicitly in the limitations that the discriminant validity between LEE and COI is borderline and that the high correlation suggests these constructs are very closely linked in their context.**

**Response:**
We sincerely thank the reviewer for this insightful observation. We fully acknowledge that the high correlation between LEE and COI (*r* = .832) and their borderline discriminant validity indicate that these constructs are closely intertwined in the context of digital textbook use.

Rather than only noting this as a limitation, we addressed the issue at the conceptual level by re-specifying our model. Guided by both theoretical reasoning and empirical evidence, LEE and COI were consolidated into a single higher-order outcome construct: Digital Textbook Usage Outcome (DTUO). This revision reflects the practical reality that, for EFL learners, perceived learning effectiveness and continuance intention represent synchronous facets of the same usage-value process.

In addition, as suggested, we explicitly discuss this measurement overlap in the revised Limitations and Future Directions section:

1.While combining LEE and COI mitigates the discriminant validity issue, reliance on self-reported measures for learning effectiveness remains a limitation that may introduce bias.

2.We recommend future research validate this consolidated construct using objective indicators (e.g., course grades, usage logs) and multi-dimensional scales to ensure stability and generalizability.

These revisions provide a clearer rationale for our model decisions and offer a more precise scholarly framing.
Revised Sections: relevant parts of the Method, Results, and Discussion sections, as well as the Limitations and Future Directions section (Pages 27–28, Lines 704–744).

**Review Comments to the Author**

**Reviewer #3:**

**Comment 1:** The study is cross-sectional; however, parts of the writing read causally (e.g., “TTF drives COI”). You do acknowledge cross-sectional limitations later, but the framing should be consistent throughout (title, abstract, discussion).

I recommend:
Replace causal verbs (“drives,” “leads to,” “results in”) with associational language (“is associated with,” “predicts,” “is linked to”) unless you provide stronger causal identification.

**Response:**

We thank the reviewer for this important methodological comment. We fully agree that, given the cross-sectional design of the study, causal inferences cannot be established.

In the revised manuscript, we have systematically revised the wording in the title, abstract, and discussion to avoid causal interpretations. Specifically, causal verbs such as “drives,” “leads to,” and “results in” have been replaced with associational language (e.g., “is associated with,” “is linked to,” and “statistically predicts”). This ensures that the framing of the findings is consistent with the correlational nature of the data.

In addition, we have clarified in the Discussion section that the observed relationships should be interpreted as statistical associations rather than causal effects, and that stronger causal identification would require longitudinal or experimental designs.

**Comment 2:** Add one short paragraph in the Methods or Discussion opening clarifying that results support the proposed directional model but do not establish causality.

**Response:**

We thank the reviewer for this helpful suggestion. We agree that the proposed directional model should not be interpreted as implying causality.

Accordingly, we have added a short paragraph at the beginning of the Discussion section to explicitly state that, although the results are consistent with the hypothesized directional relationships derived from the S–O–R framework, they do not establish causal effects. We further note that future studies employing longitudinal or experimental designs are needed to test causal directionality.

Revised Sections: Title, Abstract, Discussion, and Limitations and Future Directions

**Comment 3:** You used a single-factor CFA and conclude CMV is unlikely because the one-factor model fits poorly.
This is a very common first step, but reviewers often see it as insufficient on its own, especially when all constructs are self-reported in one survey.
Actionable improvements:
Add a marker variable approach or a latent method factor test in CFA/SEM (even as a robustness check).
At minimum, report procedural remedies more explicitly (e.g., psychological separation, anonymity wording, item randomisation, reducing evaluation apprehension), not only ethics/confidentiality.

**Response:**

Thank you for this important comment. We agree that relying solely on a single-factor CFA is insufficient when all constructs are measured via self-report in a single survey. Following the reviewer’s actionable suggestions, we have strengthened both the procedural and statistical remedies for common method variance (CMV).

(1) Procedural remedies.
We have expanded the Methodology section to report procedural controls more explicitly. In addition to standard ethics and confidentiality statements, we now clarify that the survey design incorporated psychological separation of constructs, respondent anonymity, and careful item wording to reduce evaluation apprehension and social desirability bias (Podsakoff et al., 2003).

(2) Statistical remedies (latent method factor test).
Following the reviewer’s recommendation, we conducted a latent method factor test using the Unmeasured Latent Method Construct (ULMC) approach (Liang et al., 2007) on the revised measurement model. A common latent factor was added and linked to all observed indicators, and model fit and factor loadings were compared with those of the original model.

The results showed that although the chi-square difference was significant (Δχ²(17) = 63.069, p < .001), changes in other fit indices were small (ΔCFI = 0.014, ΔTLI = 0.010, ΔRMSEA = 0.008). More importantly, differences in standardized factor loadings were minimal, with a maximum change of 0.073, well below the conservative threshold of 0.20 (Cohen, 1988).

Together, these findings indicate that CMV is unlikely to pose a serious threat to the validity of the structural relationships in the revised model. These additions have been incorporated into the revised manuscript (see Common method variance section, pages 13-14, lines 347-371).

**Comment 4:** You note that “the square root of AVE for LEE fell slightly below its correlation with a related construct,” and you conclude discriminant validity is “acceptable.”
Given the strong correlation between LEE and COI (reported as high), this needs more than a brief reassurance.
Actionable improvements:
Report HTMT (Henseler et al.) for discriminant validity. Many journals increasingly expect HTMT alongside Fornell–Larcker.
Consider whether COI and LEE are conceptually too close in your operationalisation (see next point); if so, revise measurement or acknowledge overlap and interpret cautiously.

**Response:**

Thank you for this important methodological suggestion. We agree that the high correlation between LEE and COI raises substantive concerns regarding their discriminant validity and should not be addressed through brief reassurance alone.

Following the reviewer’s recommendation, we additionally examined discriminant validity using the HTMT criterion (Henseler et al.). The HTMT value between LEE and COI exceeded the commonly recommended threshold, suggesting insufficient empirical separation between the two constructs in the present dataset.

Given this result, we adopted a conservative modeling strategy. Rather than treating LEE and COI as fully distinct latent variables, we reconsidered their specification in the measurement model. Conceptually, perceived learning effectiveness and continuance intention both capture closely related evaluative aspects of learners’ digital textbook use outcomes and may therefore be difficult to distinguish empirically in a cross-sectional, single-source survey design.

Accordingly, in the revised model we specified LEE and COI as first-order dimensions of a higher-order construct, termed Digital Textbook Usage Outcome (DTUO). This approach allows us to acknowledge their empirical overlap while retaining their theoretical distinction at the indicator level. The revised measurement model demonstrates acceptable validity, and the results are interpreted at the level of the higher-order outcome construct.

In addition, in the Limitations section, we acknowledge that the use of a brief self-report measure of perceived effectiveness and a cross-sectional design may limit the precision with which distinct outcome dimensions can be differentiated. Accordingly, future research should employ longitudinal and multi-method approaches to further disentangle perceived effectiveness and continuance intention.

**Comment 5:** LEE is measured using only three items (and appears behavioural-leaning by your description), which you also acknowledge as a limitation.

But this construct is central (largest direct effect on COI). With a brief scale, the model may be capturing “perceived progress” rather than a broader concept of effectiveness.
Actionable improvements:
Clarify exactly what LEE represents: perceived learning gains, behavioural engagement outcomes, or self-reported effectiveness.
If feasible, add at least one objective proxy (course grade, quiz performance, usage analytics) or justify why self-reported LEE is appropriate and what it can/cannot claim.
If you cannot add objective outcomes, expand the limitations with a sharper statement on self-report bias and measurement breadth.

**Response:**

Thank you for this helpful comment. We agree that the construct of learning effectiveness (LEE) requires careful clarification and cautious interpretation given its central role in the model and its brief measurement.

In the revised manuscript, we have clarified that LEE is conceptualized as perceived learning effectiveness, focusing on learners’ self-reported behavioral and engagement-related outcomes when using digital textbooks, rather than objective academic achievement. This operationalization is informed by the behavioral (Level 3) dimension of the Kirkpatrick evaluation model and primarily reflects perceived changes in learning attitudes, interest, and effort investment. The description in the Measures section has been revised accordingly to avoid implying that LEE captures comprehensive or objective learning performance.

We further acknowledge that the three-item self-report scale is limited in construct breadth and is susceptible to self-report bias. Accordingly, we have strengthened the Limitations section to state explicitly that the findings should be interpreted as referring to perceived rather than actual learning outcomes. We also note that future research should incorporate objective proxies (e.g., course grades, quiz performance, and usage logs) and more multidimensional measures to more fully capture learning effectiveness in digital textbook contexts.

### Revised Section: See Measures (Page 11, Lines 295–302), Limitations and future directions (pages 27–28, lines 716–723)

**Comment 6:** The paper suggests “emotion recognition interfaces that dynamically adjust content difficulty based on real-time learner emotion levels.”
This is a very strong claim with privacy, feasibility, and ethics implications. In the current manuscript, it appears abruptly and is not grounded in your empirical design (you did not measure emotion recognition, biometric data, or adaptive systems).
Actionable improvements:
Reframe this as a future research direction or an optional advanced design concept, not a policy requirement.
If you keep it as an implication, you need a short cautionary note about data privacy, consent, transparency, and algorithmic limitations, and cite relevant literature.

**Response:**
We thank the reviewer for this critical and constructive comment. We acknowledge that the present study examined learners’ self-reported affective states rather than real-time emotion recognition, biometric data, or adaptive systems. Therefore, the earlier wording regarding the implementation of emotion recognition interfaces exceeded the scope of our empirical design.

Following the reviewer’s recommendation, we have revised this part of the manuscript as follows:

1.We have removed the strong recommendation for real-time emotion recognition from the Practical Implications section to avoid overstating the empirical basis of our findings.

2.We have reframed this idea as a future research direction in the Future Directions section, presenting it as an optional and exploratory design concept rather than a policy or design requirement.

3.In line with the reviewer’s guidance, we have added a brief cautionary note addressing issues of data privacy, informed consent, algorithmic transparency, and ethical constraints, and emphasized that any future development of emotion-aware adaptive systems must adhere to strict ethical and governance standards.

These revisions ensure that the discussion remains aligned with the study’s empirical scope while indicating potential avenues for future investigation in a theoretically informed and ethically responsible manner.

### Revised Section: See practical implications (Page 26–27, Lines 669–703), Limitations and future directions (lines 724–736)

**Comment 7:**

Conduct a full reference audit: ensure every reference is real, correctly formatted, and has a valid DOI/URL where applicable.

**Response:**

Thank you for this valuable suggestion. We have conducted a full audit of all references in the revised manuscript. Each citation has been carefully checked to ensure that (a) the source is authentic and traceable, (b) bibliographic information (authors, year, title, journal, volume, and pages) is accurate, and (c) a valid DOI or URL is provided wherever available. All detected errors and inconsistencies have been corrected accordingly.

These revisions are reflected in the updated reference list in the revised manuscript (see References pages 29-37, lines 759-1003).

### **The review comments in the attached file**

Task-technology fit drives digital textbooks continuance: Serial mediation of positive emotion and learning effectiveness

The article showed clear problem framing. The decline in COI of digital textbooks in EFL contexts is well established. The importance of post-adoption behavior is clearly justified. This aligns well with PLOS ONE’s preference for clearly motivated applied research.

**Comment 1:** However, the articulation of contribution is somewhat weak. You repeatedly state that “few studies have examined emotions and cognition jointly” but this claim is too general. Not sufficiently bounded to EFL digital textbooks. Not explicitly contrasted with what existing studies already do. PLOS ONE requires a clearly stated incremental contribution even if modest, it must be explicit and concrete. You must clearly state what is new, in terms of contextual (EFL digital textbooks specifically), structural (serial mediation vs parallel mediation), and/ or theoretical integration (explicit S-O-R operationalization of TTF).

**Response:**

Thank you for this important comment. We agree that the study’s contribution needed to be stated more explicitly and in clearer relation to prior work. We have therefore rewritten the Theoretical Implications section to specify the study’s incremental contribution at three levels.

First, at the contextual level, this study extends task–technology fit (TTF) research to the specific setting of EFL digital textbooks, which differs from prior work mainly conducted in generic e-learning or mobile learning systems.

Second, at the structural level, instead of modeling TTF only as a direct predictor of outcomes, we embed it in a Stimulus–Organism–Response (S–O–R) framework, conceptualizing TTF as a stimulus operating through learners’ positive emotion to relate to usage outcomes.

Third, at the theoretical integration level, we refine the “Response” component by modeling learning effectiveness and continuance intention as a unified usage outcome construct, capturing both perceived effectiveness and future use intention.

These revisions clarify what is new in terms of research context, model structure, and theoretical integration, and are now stated explicitly in the Theoretical Implications section (pages 24–26, lines 626–667).

**Comment 2:** There are overlapping hypotheses (H7–H9). H7, H8, and H9 are conceptually overlapping:

- H7: POE mediates TTF → COI
- H8: LEE mediates TTF → COI
- H9: POE → LEE serial mediation

For reviewers, this can appear as hypothesis inflation. This is not incorrect, but it must be justified explicitly as, testing partial vs full mediation and/or comparing parallel vs serial mechanisms.

**Response:**
We thank the reviewer for this insightful comment regarding the conceptual overlap among the mediation hypotheses (H7–H9) in the previous version of the manuscript. We agree that, when presented without sufficient theoretical clarification, the parallel and serial mediation structure involving positive emotion (POE) and learning effectiveness (LEE) could be perceived as conceptually overlapping.

In the revised manuscript, rather than retaining multiple mediation paths, we re-specified the model based on a clearer theoretical rationale grounded in the Stimulus–Organism–Response (S–O–R) framework. Specifically, task–technology fit (TTF) is conceptualized as an environmental stimulus, positive emotion (POE) as the organismic state, and digital textbook usage outcomes (DTUO) as the response.

In developing this revised structure, we reconsidered the conceptual relationship between learning effectiveness (LEE) and continuance intention (COI) in the context of digital textbook use. Perceived learning effectiveness and continuance intention both reflect learners’ evaluations of usage outcomes and are likely to co-occur closely in practice. Based on this theoretical consideration, together with empirical evidence of their strong association, we re-specified LEE and COI as two first-order dimensions of a higher-order construct, termed Digital Textbook Usage Outcome (DTUO).

Accordingly, the mediation mechanism is now represented by a single theoretically coherent pathway (TTF → POE → DTUO). This revision eliminates conceptual redundancy among mediation hypotheses while providing a more parsimonious and theoretically consistent explanation of how task–technology fit is linked to learners’ outcomes in the context of digital textbook use. (See Hypothesis Development, pages 5–8, lines 91–205 )

**Comment 3:** Issues related to language precision and academic tone must be addressed. This includes minor grammatical issues (e.g., “learner’ s”), occasional informal phrasing (“great understanding”), inconsistent pluralization (“digital textbook” vs “digital textbooks”).

**Response:**

We thank the reviewer for pointing out these issues regarding language precision and academic tone. We have carefully revised the entire manuscript to improve grammatical accuracy, stylistic consistency, and formal academic expression. Specifically, we corrected minor grammatical errors (e.g., possessive forms such as “learner’s”), replaced informal or vague expressions (e.g., “great understanding”) with more precise academic wording, and ensured consistent use of key terms (e.g., “digital textbooks” vs. “digital textbook”) throughout the text.
These revisions were applied across the manuscript to enhance clarity, precision, and overall readability in accordance with the style requirements of PLOS ONE.

**Comment 4:** In Methods section, the description of the Output-Oriented Approach and philosophical elements (“integration of culture and Taoism”) is overly descriptive and pedagogical, not methodological. Prioritize method relevance, not curriculum exposition.

**Response:**

We thank the reviewer for this helpful comment. We agree that the previous description of the Output-Oriented Approach and related philosophical elements was overly descriptive and pedagogical for a Methods section.

In the revised manuscript, we have substantially condensed and refocused this subsection to emphasize only method-relevant information, including (a) the textbook version used, (b) its key digital functions, and (c) how the platform was implemented uniformly across participants. Descriptive content related to curricular philosophy and cultural integration has been removed.

The revised text now concentrates on ensuring transparency of the research materials and the consistency of participants’ exposure to the digital textbook environment, in line with the methodological focus required for this section. (See Method, page 8, lines 207-219)

**Comment 5:** You stated, “All scales underwent rigorous validation to ensure construct reliability and validity prior to deployment.” This is misleading. Validation occurred during analysis, not prior to deployment. This requires revision. Rephrase to: “All scales were adapted from validated instruments and subsequently assessed for reliability and validity in the present study.” This is essential to avoid methodological misrepresentation.

**Response:**

We thank the reviewer for this important clarification. We agree that our original wording was misleading. The manuscript has been revised to state that all measurement instruments were adapted from previously validated scales and that their reliability and validity were subsequently assessed in the present study, rather than prior to deployment. This revision avoids any implication that full validation occurred before data collection. (See Instrument, Scale adaptation and validation procedure, page 10, Lines 251–255)

**Comment 6:** You relied only on Harman’s single-factor CFA, which can be considered insufficient alone. Do not claim CMV is “not a problem.” Add procedural remedies already implied, anonymity, voluntary participation, and separation of constructs.

**Response:**
We thank the reviewer for this insightful comment. We agree that relying solely on Harman’s single-factor test is insufficient to rule out common method variance (CMV), and that it would be inappropriate to state that CMV is “not a problem.”

In response, we have revised the manuscript in two respects.

First, as an additional statistical check, we incorporated a latent method factor into the CFA model to examine the potential influence of CMV. The results show that introducing the method factor did not materially change the pattern or magnitude of the substantive factor loadings and structural paths, suggesting that CMV is unlikely to have substantially biased the estimated relationships in the model.

Second, we have expanded the Methods section to report the procedural remedies implemented during data collection to mitigate CMV. These include ensuring respondent anonymity, emphasizing voluntary participation, and separating key constructs within the questionnaire design to reduce evaluation apprehension and consistency motifs.

In addition, we have revised the wording throughout the manuscript to avoid absolute claims about the absence of CMV and instead state that no serious CMV threat was detected based on both procedural and statistical assessments.

These revisions have been incorporated into the Common Method Variance section (see Pages 13–14, Lines 347–371).

**Comment 7:** The high correlation between COI and LEE (0.832) may imply multicollinearity which is vital in SEM. Present VIF results and address possible concern.

**Response:**

Thank you for this important methodological suggestion. We agree that the high correlation between LEE and COI raises substantive concerns regarding their discriminant validity and should not be addressed through brief reassurance alone.

Following the reviewer’s recommendation, we additionally examined discriminant validity using the HTMT criterion (Henseler et al.). The HTMT value between LEE and COI exceeded the commonly recommended threshold, suggesting insufficient empirical separation between the two constructs in the present dataset.

Given this result, we adopted a conservative modeling strategy. Rather than treating LEE and COI as fully distinct latent variables, we reconsidered their specification in the measurement model. Conceptually, perceived learning effectiveness and continuance intention both capture closely related evaluative aspects of learners’ digital textbook use outcomes and may therefore be difficult to distinguish empirically in a cross-sectional, single-source survey design.

Accordingly, in the revised model we specified LEE and COI as first-order dimensions of a higher-order construct, termed Digital Textbook Usage Outcome (DTUO). This approach allows us to acknowledge their empirical overlap while retaining their theoretical distinction at the indicator level. The revised measurement model demonstrates acceptable validity, and the results are interpreted at the level of the higher-order outcome construct.

In addition, in the Limitations section, we acknowledge that the use of a brief self-report measure of perceived effectiveness and a cross-sectional design may limit the precision with which distinct outcome dimensions can be differentiated. Accordingly, future research should employ longitudinal and multi-method approaches to further disentangle perceived effectiveness and continuance intention.

(Pages 17–18, Lines 438–456).

**Comment 8:** It was stated that, “The square root of AVE for LEE fell slightly below its correlation…” This technically violates Fornell–Larcker. What is your solution on this?

**Response:**

We thank the reviewer for highlighting this technical issue. As noted, the square root of AVE for LEE fell slightly below its correlation with COI, indicating potential discriminant validity concerns under the Fornell–Larcker criterion.

To address this, we reconsidered the conceptual relationship between LEE and COI in the context of digital textbook use. Perceived learning effectiveness (LEE) and continuance intention (COI) both represent learners’ evaluations of usage outcomes and are expected to co-occur closely in practice. Based on this theoretical reasoning and empirical evidence, we re-specified LEE and COI as first-order dimensions of a higher-order construct, Digital Textbook Usage Outcomes (DTUO). This modification resolves the discriminant validity issue and better reflects the intertwined nature of learning effectiveness and continuance intention in the EFL digital textbook context.

**Comment 9:** Discussion is repetitive and descriptive, with limited abstraction. Condense repetitive confirmations and elevate synthesis. Focus more on mechanisms, less on restating coefficients. Emphasize why LEE dominates COI.

**Response:**

We thank the reviewer for the constructive feedback regarding the discussion section. In response, we have revised the discussion to reduce repetitiveness and descriptive content, and to elevate the level of abstraction. Specifically, we focused more on explaining the underlying mechanisms through which task–technology fit (TTF) influences digital textbook usage outcomes (DTUO) via positive emotion (POE), rather than reiterating the statistical results or coefficients.

We also note the reviewer’s suggestion to emphasize why learning effectiveness (LEE) dominates continuance intention (COI). In the revised manuscript, the conceptual model has been re-specified, with LEE and COI integrated as two first-order dimensions of a higher-order DTUO construct. As a result, the discussion no longer frames LEE and COI as separate, and thus the notion of one “dominating” the other is no longer applicable. Our discussion now emphasizes the joint role of these dimensions in reflecting learners’ holistic evaluations of digital textbook use, in line with the theoretical rationale of the S–O–R framework. (See Discussion pages 20–24, lines 507–624)

**Comment 10:** The Pedagogical & Policy Implication section overreaches empirically, especially, “…emotion recognition interfaces that dynamically adjust content difficulty…” Your study did not measure emotion recognition technology. Reframe as speculative future directions, not implications.

**Response:**
We thank the reviewer for highlighting the overreach regarding emotion recognition interfaces. We acknowledge that our study measured self-reported affective states rather than real-time biometric or algorithmic emotion recognition, and therefore, any suggestion for implementing such systems as a policy implication exceeds the empirical support. Following the reviewer’s advice, we have reframed this concept as a future research direction rather than a current practical recommendation. Specifically:

(1)We have removed the strong recommendation for real-time emotion recognition from the “Pedagogical & Policy Implications” section to avoid overstating our findings.

(2)The concept is now discussed in the Future Directions section, with a cautionary note emphasizing the need for ethical standards, data privacy, user consent, and algorithmic transparency in any future exploration of adaptive emotional systems.

This revision ensures that our manuscript accurately reflects the scope of our empirical evidence while acknowledging potential avenues for future research.

### Revised Section: See practical implications (Page 26–27, Lines 669–703), Limitations and future directions (lines 724–736)
